# Synergism of Antimicrobial Frog Peptides Couples to Membrane Intrinsic Curvature Strain

**DOI:** 10.1016/j.bpj.2018.03.006

**Published:** 2018-04-25

**Authors:** Regina Leber, Michael Pachler, Ivo Kabelka, Irene Svoboda, Daniel Enkoller, Robert Vácha, Karl Lohner, Georg Pabst

**Affiliations:** 1 Institute of Molecular Biosciences, Biophysics Division, University of Graz, NAWI Graz, Graz, Austria; 2 BioTechMed Graz, Graz, Austria; 3 Central European Institute of Technology, Brno, Czech Republic; 4 Faculty of Science, Masaryk University, Brno, Czech Republic; 5 Fresenius Kabi Austria GmbH, Graz, Austria

## Abstract

Mixtures of the frog peptides magainin 2 and PGLa are well-known for their pronounced synergistic killing of Gram-negative bacteria. We aimed to gain insight into the underlying biophysical mechanism by interrogating the permeabilizing efficacies of the peptides as a function of stored membrane curvature strain. For Gram-negative bacterial-inner-membrane mimics, synergism was only observed when the anionic bilayers exhibited significant negative intrinsic curvatures imposed by monounsaturated phosphatidylethanolamine. In contrast, the peptides and their mixtures did not exhibit significant activities in charge-neutral mammalian mimics, including those with negative curvature, which is consistent with the requirement of charge-mediated peptide binding to the membrane. Our experimental findings are supported by computer simulations showing a significant decrease of the peptide-insertion free energy in membranes upon shifting intrinsic curvatures toward more positive values. The physiological relevance of our model studies is corroborated by a remarkable agreement with the peptide’s synergistic activity in *Escherichia coli*. We propose that synergism is related to a lowering of a membrane-curvature-strain-mediated free-energy barrier by PGLa that assists membrane insertion of magainin 2, and not by strict pairwise interactions of the two peptides as suggested previously.

## Introduction

Antimicrobial peptides (AMPs) are highly effective components of the innate immune system of most living organisms. AMPs respond to invading pathogens and are optimized to kill bacteria either by direct interaction or by immunomodulatory activities (1, 2, 3). Ever since the discovery of AMPs in prokaryotes in the 1930s and a few decades later in eukaryotes, research efforts devoted to the molecular mechanisms of their direct antimicrobial activities have revealed several modes of interaction (for review, see, e.g., (4, 5)). A general consensus has been reached in recognizing that the positive charge of the peptide is essential for initial binding to the anionic bacterial membrane surface, a factor which allows discrimination between bacterial and host cell membranes, whereas hydrophobicity is needed for insertion into and disruption of the target membrane (see, e.g., (6, 7)). Magainin 2 (MG2) and PGLa, two members of the magainin family isolated from the skin of the African clawed frog *Xenopus laevis* (8, 9), are among the best-studied AMPs. They exhibit a broad spectrum of activity against microorganisms with minimal inhibitory concentration (MIC) values ranging from 5 to >100 *μ*g/mL (10). Of particular interest is their synergistic activity, which has been observed in bacteria but also in lipid-only model membranes (11, 12, 13, 14, 15, 16). Notably, MG2/PGLa synergism is most pronounced for Gram-negative strains such as *Escherichia coli* (13).

Unraveling the molecular origin of this synergistic activity has not been without disparity. Early biophysical studies on lipid-only systems suggested enhanced membrane-pore-formation capabilities of equimolar peptide mixtures (13, 14, 15). Indeed, a stable transmembrane orientation of PGLa/MG2 dimers, as reported from solid-state NMR measurements, supported this model (17). A few years later, however, Salnikov and Bechinger showed that the topology of PGLa and MG2 (i.e., their orientation with respect to the bilayer surface), and in particular that of MG2, depends on the hydrophobic thickness of the lipid bilayer (18). The important role of membrane physical parameters was further emphasized by Strandberg et al. (19) by correlating the topology of both peptides with the intrinsic curvature of lipids. In particular, these authors suggested that the synergistic insertion of MG2 and PGLa into membranes couples to a positive intrinsic curvature.

**Figure 1:**
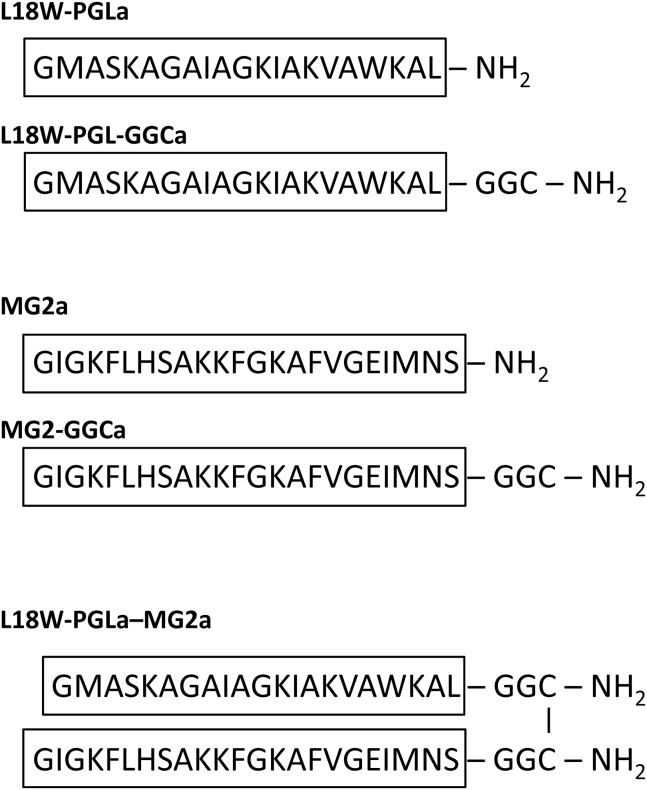
Primary structures of studied magainins.

Intriguingly, however, cytoplasmic membranes of Gram-negative bacteria are enriched in lipids with negative intrinsic curvature, such as phosphatidylethanolamine (PE) (20). This prompted us to correlate membrane-permeabilizing activities of magainin monomers and dimers in different lipid-only model systems with various intrinsic curvatures *J*_*0*_ determined from small-angle x-ray scattering (SAXS). In particular, we studied the activity of L18W-PGLa and MG2a, as well as their analogs containing GGC linkers, which were used to form the L18W-PGLa-MG2a hybrid peptide (Fig. 1). The choice of L18W-PGLa is motivated to facilitate better comparison to a previous study on magainin heterodimers (15). Note that the dye release from lipid vesicles induced by L18W-PGLa is almost identical to that induced by native PGLa (13). Thus, our results can be generalized to PGLa in our comparison to other reports. Likewise, MG2a is discussed synonymously with MG2.

The choice of lipid-only mimics is delicate. In addition to the PE enrichment, cytoplasmic membranes of Gram-negative bacteria also contain anionic phospholipids such as phosphatidylglycerol (PG) and cardiolipin (CL). The relative abundance of anionic lipids strongly depends on the strain (20) and growth conditions (21). For instance, the inner membrane of *Pseudomonas cepacia* contains 82% PE and 18% PG (wt% of total phospholipid), whereas *E. coli* also contains ∼10 wt% CL (20). The focus of the present work is on the role of intrinsic curvature strain in magainin synergism. Because the sign of *J*_*0*_ of CL strongly depends on the ionic composition of the aqueous buffer (22), we decided to mimic the inner membrane of Gram-negative bacteria to first order with a binary lipid mixture of POPE/POPG 3:1 mol/mol to avoid any ambiguities resulting from this issue. Additionally, phosphatidylcholine (PC)/cholesterol (Chol) mixtures served as a simple first-order model of human erythrocyte membranes considered as archetypes of mammalian plasma membranes. Again, PC/Chol mimics do not capture the full compositional complexity of such plasma membranes, but serve here to address the role of intrinsic curvature in a system lacking electrostatic interactions between anionic lipids and cationic peptide residues. Our results provide evidence that PGLa/MG2 synergism is tightly coupled to negative lipid intrinsic curvature and to negative surface charge of membranes but does not necessarily require, nor exclude, the formation of PGLa/MG2 pairs. Although electrostatic interactions between AMPs and lipid headgroups are crucial for significantly long retention time (binding) of the peptides to the bilayer surface, lipid intrinsic curvatures contribute to an activation free-energy barrier ΔG^‡^ for membrane insertion of the peptides. We propose that PGLa, because of its higher affinity for the hydrophobic core of the bilayer, lowers ΔG^‡^ for MG2 by reducing the curvature-mediated tension at the polar-apolar membrane interface and thereby potentiates the effect of MG2.

## Materials and Methods

### Lipids, peptides, and chemicals

Palmitoyl-oleoyl-phosphatidylethanolamine (POPE), dioleoyl-phosphatidylethanolamine (DOPE), palmitoyl-oleoyl-phosphatidylglycerol (POPG), palmitoyl-oleoyl-phosphatidylcholine (POPC), and 1-palmitoyl-2-hydroxy-PE (lyso-PE) were purchased from Avanti Polar Lipids, Alabaster, AL (purity >99%) as powder. Cholesterol, *cis*-9-tricosene, and Triton X-100 were obtained from Sigma-Aldrich, Vienna, Austria. L18W-PGLa and MG2a, as well as their analogs containing GGC linkers and L18W-PGLa-MG2a heterodimers, were obtained in lyophilized form (purity >95%) from PolyPeptide Laboratories (San Diego, CA). ANTS (8-aminonaphthalene-1,3,6-trisulfonic acid, disodium salt) and DPX (p-xylene-bis-pyridinium bromide) were purchased from Molecular Probes (Eugene, OR). Chemicals used for sodium dodecyl sulfate polyacrylamide gel electrophoresis (SDS-PAGE) as well as antibiotics (ampicillin sodium salt, gentamicin sulfate solution), 1,4-dithiothreitol (DTT), and Mueller-Hinton broth were obtained from Carl Roth (Karlsruhe, Germany). The Ultra-Low Range Marker for SDS-PAGE was purchased from Sigma-Aldrich.

Lipid stock solutions for sample preparation were prepared in organic solvent. In particular, lyso-PE was dissolved in chloroform/methanol/water 65:35:8 (v/v/v), whereas all other lipids, including *cis*-9-tricosene, were dissolved in chloroform/methanol (9:1; v/v). Peptide stock solutions were prepared in 0.01% acetic acid, and aliquots of the stock solutions were stored in silanized glass tubes at −20°C until use.

### Methods

Large unilamellar vesicles (LUVs) of ∼100 nm size were prepared and assayed for AMP-induced fluorescence leakage as described previously (23). Intrinsic lipid curvatures were determined using a SAXSpace small-angle x-ray camera (Anton Paar, Graz, Austria) applying previously developed procedures (24). Monte Carlo (MC) simulations were performed using the Metropolis scheme and computationally efficient implicit-solvent coarse-grained models. Lipids were described by a three-bead model developed by Cooke and Deserno (25), and the peptide was modeled by a patchy spherocylinder (26). Free-energy calculations for peptide translocation were performed using the Wang-Landau method (27). For details of experimental and simulation procedures, see the Supporting Material.

## Results

### Intrinsic curvatures of biomimetic membranes

We first determined estimates for *J*_*0*_ of the studied lipids and their mixtures using SAXS as detailed in (24) (see also Supporting Material and Fig. S1 for x-ray data). Intrinsic curvatures relate to the elastic curvature energy stored in lipid membranes (see, e.g., (28)). The following lipid mixtures are relevant for the present study: POPE/POPG (3:1, mol/mol) as a mimic of the inner membrane of Gram-negative bacteria (29), and POPC/POPG (3:1, mol/mol) as an alternative and frequently used lipid-only model of bacterial membranes (see, e.g., (18, 19)). Based on the intrinsic curvatures of the individual lipids (Table S2), we calculate, assuming linear additivity, a significantly negative value for POPE/POPG ($J_{0}^{mix}=-0.26$ nm^−1^), whereas POPC/POPG is found to have $J_{0}^{mix}∼0$ (Table 1). To investigate the role of intrinsic curvature, we further studied lyso-PE/POPE/POPG (1.6:1.4:1, mol/mol/mol), which has an overall 3:1 PE/PG molar ratio but $J_{0}^{mix}∼\;0$. Two charge-neutral mimics of mammalian membranes were considered for testing the role of Coulomb interactions in addition to the effect of stored energy strain. Our choice was to use POPC and its 3:1 (mol/mol) mixture with cholesterol, which, because of the negative intrinsic curvature of cholesterol (24), has $J_{0}^{mix}=-0.14$ nm^−1^, which served as a first-order approximate to mammalian plasma membranes.

**Table 1:** **Intrinsic Curvatures**$J_{0}^{mix}$ of Membrane Mimetics and Peptide Synergy *Σ*

Mimic (molar ratio)	$J_{0}^{mix}$ [nm^−1^]	*Σ*
POPE:POPG (3:1)	−0.258 ± 0.013	0.4 ± 0.1
POPC/POPG (3:1)	−0.012 ± 0.015	0.8 ± 0.2
lyso-PE/POPE/POPG (1.6:1.4:1)	−0.044 ± 0.059	1.3 ± 0.3
POPC	−0.022 ± 0.010	2.2 ± 0.4
POPC:Chol (3:1)	−0.140 ± 0.011	1.2 ± 0.3

For definition of *Σ*, see Eq. 1.

### Effect of intrinsic curvature and membrane charge

**Figure 2:**
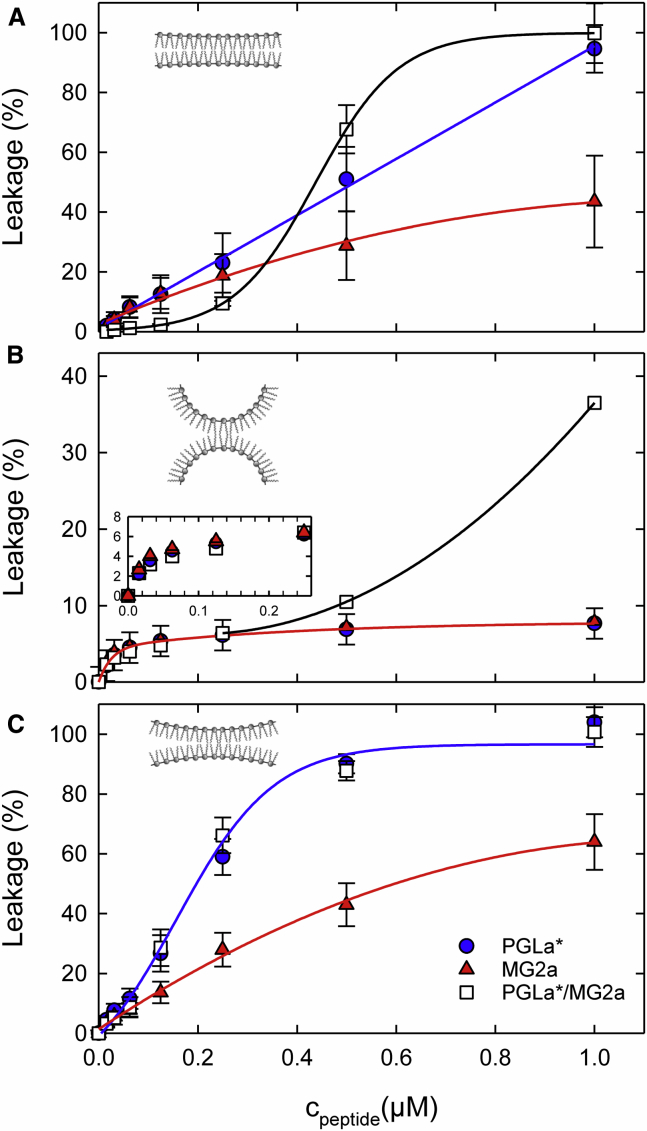
Leakage of POPC/POPG (3:1 mol/mol) (*A*), POPE/POPG (3:1 mol/mol) (*B*), and lyso-PE/POPE/POPG (1.6:1.4:1 mol/mol/mol) (*C*) LUVs as a function of L18W-PGLa and MG2a concentration, as well as their equimolar mixture. The lipid concentration was 50 *μ*M. Experimental uncertainties are determined from three independent measurements. The solid lines are guides to the eye. The membrane insets in each panel represent the monolayer curvatures as determined in Table 1. To see this figure in color, go online.

In the next step, we determined ANTS/DPX leakage in LUVs induced by L18W-PGLa, MG2a, and their equimolar mixture to exploit the effect of $J_{0}$ on AMP activity. The L18W-PGLa/MG2a mixture is motivated by previous studies (13) suggesting a pairwise interaction of the peptides to achieve synergism. In POPC/POPG ($J_{0}^{mix}∼\;0$), we found significant peptide activities (Fig. 2 *A*). Specifically, MG2a and L18W-PGLa were similarly permeabilizing vesicles at low concentrations, whereas L18W-PGLa was more effective than MG2a for *c*_*peptide*_ ≳ 0.3 *μ*M. The equimolar L18W-PGLa/MG2a mixture, in turn, was less active than the peptides individually below that concentration, suggesting some antagonizing effect of unknown origin, but one which was reproduced in several replicas. The focus of the present report is at higher concentrations, however. Here, the peptide mixture was more active than L18W-PGLa and MG2a alone for *c*_*peptide*_ ≳ 0.4 *μ*M. At *c*_*peptide*_ = 1 *μ*M, leakage induced by just L18W-PGLa was within an experimental resolution equal to that of L18W-PGLa/MG2a.

In POPE/POPG vesicles, in turn, all studied combinations of peptides caused identical leakage (≤6%) up to *c*_*peptide*_ ≈ 0.25 *μ*M and remained at the same level for the individual peptides at higher concentrations (Fig. 2 *B*). The peptide mixture, however, exhibited a strong increase of efficacy and was about six times more active than L18W-PGLa and MG2a alone at 1 *μ*M. To see whether the different AMP activities are related to intrinsic curvature, we repeated the leakage assay for lyso-PE/POPE/POPG, which has the same lipid headgroup composition as POPE/POPG but $J_{0}^{mix}∼\;0$, just as POPC/POPG. Indeed, the peptides regained membrane-permeabilizing efficacy, with L18W-PGLa and L18W-PGLa/MG2a causing full LUV leakage at 1 *μ*M concentration, just as observed for POPC/POPG (Fig. 2 *C*).

**Figure 3:**
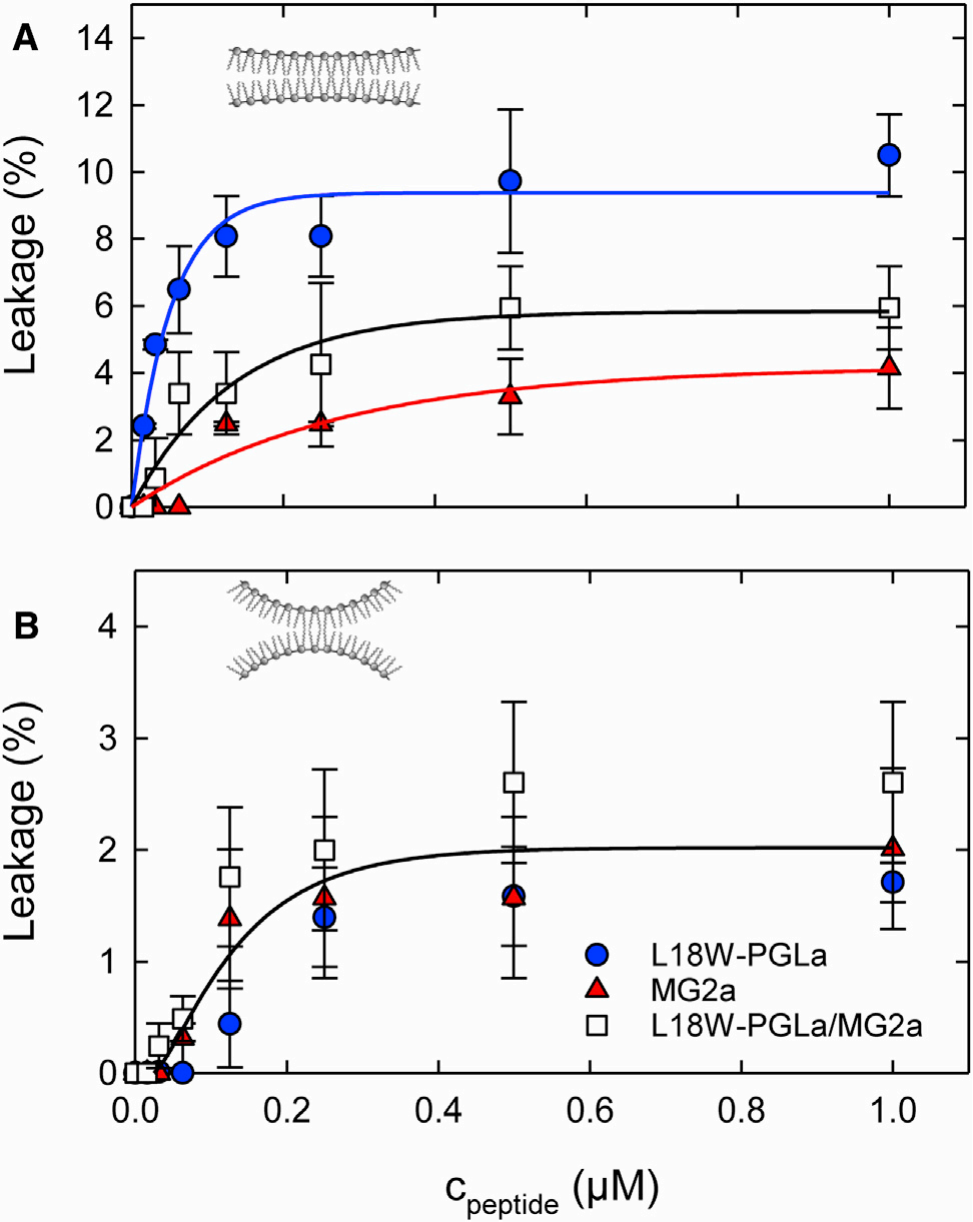
Leakage of POPC (*A*) and POPC/Chol (3:1 mol/mol) (*B*) LUVs as a function of L18W-PGLa, MG2a, and their equimolar mixture. The lipid concentration was 50 *μ*M. Experimental uncertainties are determined from three independent measurements. The solid lines are guides to the eye. The membrane insets in each panel represent the monolayer curvatures as determined in Table 1. To see this figure in color, go online.

For mammalian mimics, in turn, our experiments revealed low activity for all peptides. L18W-PGLa exhibited the highest activity, although its maximal leakage did not exceed 10% within the studied concentration range for POPC (Fig. 3 *A*). The activity of the L18W-PGLa/MG2a was between that of L18W-PGLa and MG2a at all concentrations. Most likely this is related to the reduced number of L18W-PGLa molecules in the peptide mixture. Introducing cholesterol, which shifts $J_{0}^{mix}$ to negative values, did not lead to an increase of activity of L18W-PGLa/MG2a relative to the peptide monomers as in POPE/POPG. Instead, the overall leakage dropped for all peptides almost to the noise level of the measurement (Fig. 3 *B*).

The diverse activities of the presently studied peptides can be summarized by the definition of a synergy parameter. For antimicrobial activity, this is usually based on the MIC (30). In analogy, we define

(1) $$\Sigma =\frac{L_{L18W-PGLa}+L_{MG2a}}{L_{L18W-PGLa/MG2a}},$$

where *L*_*L18W-PGLa*_ and *L*_*MG2a*_ are the leakage values for the individual peptides at half of the peptide concentration used to determine the leakage *L*_*L18W-PGLa/MG2a*_ of their mixture. Hence, *Σ* < 0.5 if both peptides act synergistically. Table 1 lists the results for the highest peptide concentration measured. Except for POPE/POPG, all model systems exhibited *Σ* > 0.5. This is consistent with a recent report on an increase of PGLa/MG2 synergy for PE/PG mixtures as compared to PC/PG (31). We thus conclude that L18W-PGLa/MG2a act synergistically only in POPE/POPG.

### Effect of peptide dimerization

**Figure 4:**
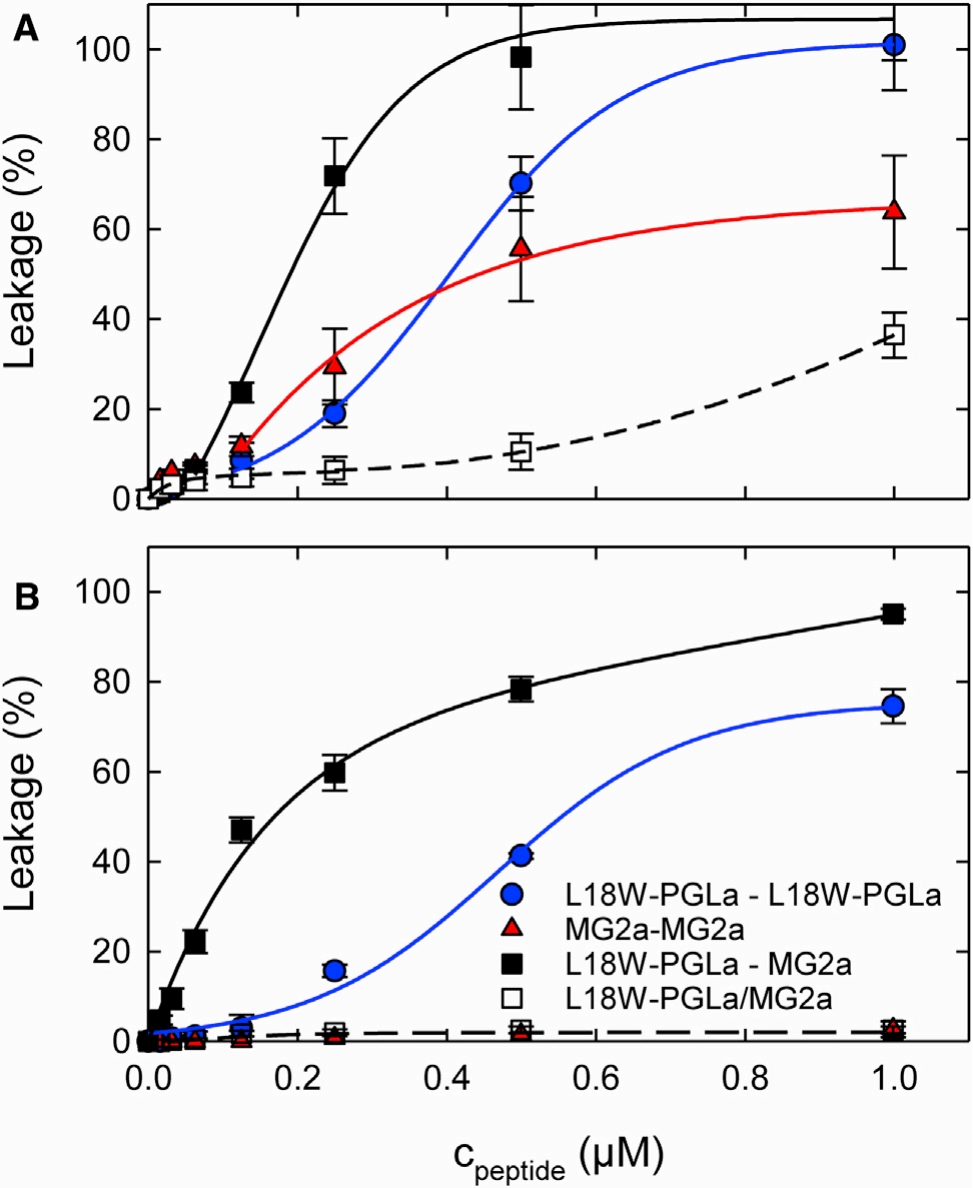
Dye release induced by L18W-PGLa/MG2a homodimers and heterodimers. (*A*) compares the activities of L18W-PGLa-L18W-PGLa and MG2a-MG2a homodimers, L18W-PGLa-MG2a heterodimers, and L18W-PGLa/MG2a equimolar mixtures in POPE/POPG LUVs. (*B*) presents results for the same peptides in POPC/Chol LUVs. Note that the results for MG2a homodimers and L18W-PGLa/MG2a are identical within experimental resolution. The lipid concentration was 50 *μ*M. The lines are guides to the eye. To see this figure in color, go online.

Synergistic effects of L18W-PGLa and MG2a have been proposed to result from pairwise peptide interaction in membranes (13). Following (16), we therefore constructed L18W-PGLa-MG2a heterodimers using L18W-PGL-GGCa and MG2-GGCa (Fig. 1). Further, L18W-PGL-GGCa and MG2-GGCa form in buffer solution (pH ∼7) homodimers (15) as verified by SDS-PAGE (Fig. S2). This enabled us to compare the activities of the diverse magainin dimers. Fig. 4 *A* shows results from leakage experiments on POPE/POPG vesicles. In general, all dimers were able to permeabilize lipid bilayers more strongly than the equimolar mixture of L18W-PGLa and MG2a. More specifically, homodimers of L18W-PGLa and MG2a exhibited similar activities for *c*_*peptide*_ < 0.1 *μ*M. At higher peptide concentrations, homodimers of L18W-PGLa exhibited a sigmoidal increase reaching nearly 100% leakage, whereas the activity of MG2a homodimers saturated at much lower leakage values (∼60%). Turning to L18W-PGLa-MG2a heterodimers, we observed leakage superior to L18W-PGLa and MG2a homodimers already at low peptide concentration. Final leakage values at *c*_*peptide*_ = 1 *μ*M were comparable to those of L18W-PGLa homodimers, however.

In the POPC/Chol mixture, effects were significantly different (Fig. 4 *B*). L18W-PGLa-MG2a heterodimers successfully permeabilized the LUVs and induced close to 100% leakage at 1 *μ*M peptide concentration. L18W-PGLa homodimers were active above concentrations of 0.1 *μ*M but reached only ∼74% leakage at the highest concentration studied. In contrast, MG2a homodimers, just like L18W-PGLa/MG2a equimolar mixtures, were not able to induce significant dye release from the vesicles. Finally, we performed a control experiment on POPE/POPG vesicles by adding DTT (40 *μ*M), which is known to reduce disulfide bonds (32), to the L18W-PGLa-L18W-PGLa solution before mixing the peptides with LUVs*.* This in situ transformation of L18W-PGLa homodimers to monomers resulted in permeabilization capabilities identical with that of L18W-PGLa peptide (Fig. S3). Thus, the membrane-lytic activity of L18W-PGLa can be increased strongly by chemical fixation as dimers.

### Free energy of peptide translocation

**Figure 5:**
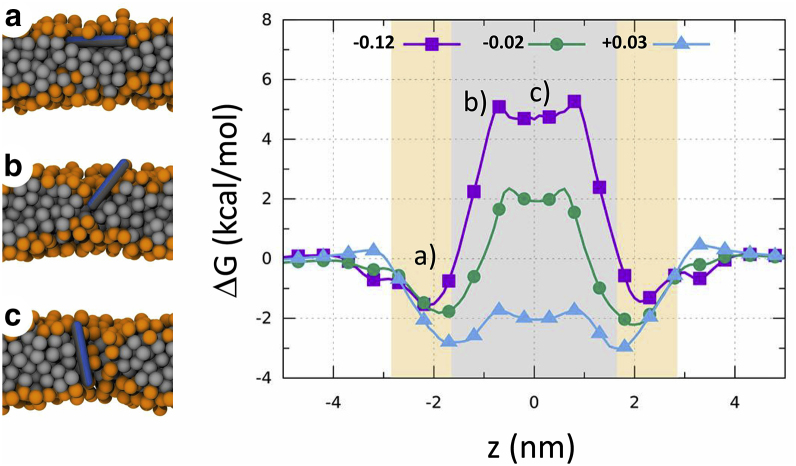
Calculated free energy profiles of peptide translocation across membranes with different intrinsic curvature (numbers in legend correspond to *J*_*0*_ in nm^−1^). The headgroup and tail regions are shown as orange and gray areas, respectively. The left-hand side shows simulation screen shots of the most important steps of peptide translocation: (*a*) surface adsorption, (*b*) peptide tilting, and (*c*) peptide insertion. To see this figure in color, go online.

To get deeper insight on the influence of intrinsic curvature on peptide insertion, we performed coarse-grained MC simulations on the translocation of an MG2a mimic through lipid bilayers of different *J*_*0*_. The sign of intrinsic curvatures of the simulated systems corresponded to those of the measured systems and also included a system of positive intrinsic curvature (see Supporting Material). Simulations clearly show that membrane composition affects the free-energy profiles (Fig. 5). In particular, the system with the most negative *J*_*0*_ exhibits the highest free-energy penalty for translocation of the peptide across the membrane. The barrier decreases significantly with shifting *J*_*0*_ toward positive values, which makes the peptide translocation much easier. The translocation is mainly determined by the insertion step, which requires a change in orientation from a parallel to perpendicular/tilted peptide alignment with respect to the membrane surface. Note that the free-energy minimum corresponds to the peptide position in the membrane adsorbed state, which shifts progressively toward the membrane center with increasing *J*_*0*_, i.e., positive curvature.

### Antimicrobial activity of peptide monomers and dimers against *E. coli*

**Figure 6:**
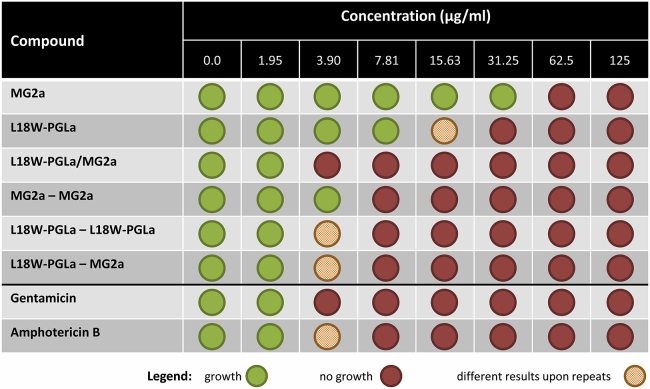
Effect of different magainins on the growth of *E. coli* K12. The antibiotics gentamicin and amphotericin B serve as positive controls. To see this figure in color, go online.

To correlate our results on membrane mimetics to biological activities toward bacteria, we determined the effect of peptides on *E. coli* growth. We observed distinct MIC values for the individual peptides (Fig. 6). In agreement with (16, 31), we found synergistic activity of L18W-PGLa/MG2 mixtures (Σ∼0.25) as compared to single-peptide applications. Further, chemically fixed peptide homodimers were significantly more active than their monomers. MG2a dimers reduced the MIC from 62.5 to 7.8 *μ*g/mL and L18W-PGLa dimers from ∼31.25 to ∼7.8 *μ*g/mL, respectively. In particular, L18W-PGLa homodimers exhibited the same efficacy as L18W-PGLa-MG2a heterodimers.

The MIC values can be compared qualitatively to our dye-release experiments on vesicles using the leakage intensities observed at the highest-measured peptide concentrations. Notably, the synergistic activity of the equimolar peptide mixture (Fig. 2 *B*), the similar activities of L18W-PGLa homodimers and L18W-PGLa-MG2a (Fig. 4 *A*), and the high efficacy of L18W-PGLa homodimers compared to individual L18W-PGLa peptide applications (Fig. S3) are well-captured by the POPE/POPG (3:1 mol/mol) mimic. Other features, such as the different antimicrobial activities of L18W-PGLa and MG2, as well as the almost equal efficacy of equimolar peptide mixtures and L18W-PGLa-MG2a hybrids, are not observed in our simple *E. coli* inner membrane mimics, which may be explained by the vastly more complex structure of the *E. coli* cell envelope that consists of an outer membrane, periplasm, and inner membrane. We emphasize, however, that the synergistic activity of L18W-PGLa and MG2a, which is the main focus of this work, is reproduced in POPE/POPG 3:1 (mol/mol).

We can further compare our leakage results on POPC/Chol vesicles to the peptide’s hemolytic activity. Nishida et al. (16) reported a sixfold increase of erythrocyte hemolysis for PGLa-MG2a heterodimers as compared to the PGLa/MG2a equimolar mixture. Further, compared to single peptides, the heterodimer’s hemolytic activity was even 40- to 60-fold increased. This latter result correlates qualitatively with our findings in POPC/Chol (3:1 mol/mol) vesicles, in which the heterodimers showed a 50 times higher activity than the L18W-PGLa/MG2a mixture (Fig. 4 *B*). Hence, POPC/Chol appears to be a reasonable first-order lipid-only model to estimate the hemolytic potential of magainins.

## Discussion

### Synergism is coupled to negative intrinsic curvature

Coupling experimental data for intrinsic curvatures and AMP-induced ANTS/DPX dye release from selected lipid-only mimics of bacterial and mammalian membranes provided clear evidence for the delicate balance of membrane curvature elasticity, overall bilayer surface charge, and peptide properties in promoting or obstructing the synergistic activity of L18W-PGLa/MG2a mixtures. The synergism of PGLa and MG2 has been studied before by leakage assays on diverse lipid vesicles, showing significant enhancement of peptide activity for PGLa/MG2 equimolar mixtures (13, 33).

Pronounced synergism was observed only in POPE/POPG bilayers (Fig. 2 *B*). The requirement of high peptide concentration to permeabilize PE-enriched bilayers may be additionally enhanced by extensive intermolecular H-bonding of PEs (34, 35). Most interestingly, however, L18W-PGLa/MG2a synergism is fully abolished in lyso-PE/POPE/POPG (Fig. 2 *C*). This system was designed to have 1) the same overall charge as POPC/POPG and POPE/POPG, 2) the same headgroup composition as POPE/POPG (i.e., the same abilities to form H-bonds), and 3) the overall same intrinsic curvature as POPC/POPG (J_0_∼0). The loss of synergism in lyso-PE/POPE/POPG hence provides unambiguous evidence for a coupling to negative intrinsic curvature.

It has been recognized for some time that protein/peptide activity couples to intrinsic lipid curvatures (36, 37, 38). Intrinsic lipid curvature is a property of lipid monolayers (i.e., each membrane leaflet) that gives rise to an elastic energy stored in flat lipid bilayers (see, e.g., (39)), which may be released upon interaction with a membrane active compound (36). That is, lateral strain, in particular at the polar/apolar interface, is significantly larger for lipids with a more negative *J*_*0*_, whereas lipid membranes with a positive *J*_*0*_ have a looser polar/apolar interface. Membrane intrinsic curvature has been previously related to MG2 activity (40) or synergism (19). Conversely, the latter authors concluded that synergistic interactions between PGLa/MG2 are related to positive *J*_*0*_ that would allow the peptides to penetrate into the bilayer more easily (19). This assessment was based on the assumption that synergism is related to the adoption of a tilted or inserted PGLa topology in the presence of MG2. This hypothesis has been cast into doubt by (41, 42), which show that the peptides develop synergistic activities in an in-planar topology. Strandberg et al. (19) also notably reported mainly surface aligned PGLa/MG2 for POPC/POPG bilayers, whereas MG2-mediated PGLa insertion was reported only for lipid mixtures with disaturated hydrocarbon chains (17, 19).

Based on our results, we agree with (17, 19) that membranes with a more positive curvature facilitate peptide insertion into the bilayer interface because of reduced lateral stress at the bilayer’s polar/apolar interface (i.e., looser packing). However, at the same time, this hampers the development of synergy because both magainins are able to disrupt the polar/apolar interface at low energetic cost. POPE/POPG (3:1 mol/mol), in turn, displays substantial interfacial stress, and indeed both magainins did not induce significant vesicle leakage (Fig. 2 *B*). However, when applied as an equimolar mixture, they clearly showed synergy with *Σ* = 0.4 (Table 1), which is in good agreement with recent observations from Zerweck et al. (31). Corroborated by our MC simulations (Fig. 5), these findings strongly suggest an activation barrier for vesicle leakage, $\Delta G^{‡}$, that is mediated by the interfacial packing density and hence lipid composition. In addition, synergy requires anionic lipids for increased affinity of peptides to the membrane surface. This is clearly demonstrated by POPC/Chol, which contains significant intrinsic negative curvature energies but resulted in a synergy parameter *Σ* = 1.2 (Table 1).

To appreciate the different activities of L18W-PGLa and MG2a, it is instructive to consider the energetics of membrane insertion based on the Wimley-White hydrophobicity scale (43). We thus calculated estimates for the Gibbs free energy of insertion into the bilayer nonpolar region (44)

(2) $$\Delta G_{ins}^{o}\approx \Delta G_{f}^{o}+\Delta G_{oct}^{o}-\Delta G_{if}^{o}\approx \Delta G_{oct}^{o}-\Delta G_{if}^{o}=\Delta G_{oct-if}^{o},$$

using the Membrane Protein Explorer (MPEx, http://blanco.biomol.uci.edu/mpex/), where $\Delta G_{f}^{o}$ is the free energy from peptide conformational changes in the aqueous phase (assumed to be insignificant), $\Delta G_{oct}^{o}$ is the free energy of peptide transfer from water to octanol, and $\Delta G_{if}^{o}$ is the free energy of binding to the membrane-water interface. Calculation of $\Delta G_{if}^{o}$ requires knowledge of the peptide’s helicity taken up at the membrane surface. For charged membranes, electrostatic interactions need to be considered (45). However, corrections calculated via the Gouy-Chapman theory for melittin yielded the same surface-partition coefficient for charged and charge-neutral bilayers (45). For our semiquantitative discussion, it thus suffices to derive $\Delta G_{if}^{o}$ based on the peptide’s helicity in PC bilayers (Table 2). Our $\Delta G_{ins}^{o}$ estimates show that L18W-PGLa has a higher propensity for being located within the membrane core than MG2a. This agrees with previous NMR data showing that PGLa may tilt into the bilayer core, whereas MG2 always remained parallel at the membrane surface (18, 19).

**Table 2:** Thermodynamics of AMP Insertion in PC Membranes According to the Wimley-White Hydrophobicity Scale

	% helix in PC	$\Delta G_{if}^{o}$(kcal/mol)	$\Delta G_{oct}^{o}$(kcal/mol)	$\Delta G_{ins}^{o}$(kcal/mol)	$\mu_{H}\;$
MG2a	83^a^	−7.3	16.9	24.2	14.1
L18W-PGLa	72^b^	−6.1	16.2	22.3	10.9

The Wimley-White hydrophobicity scale is from Ref. (43).

a Taken from (66).

b Taken from (67).

**Figure 7:**
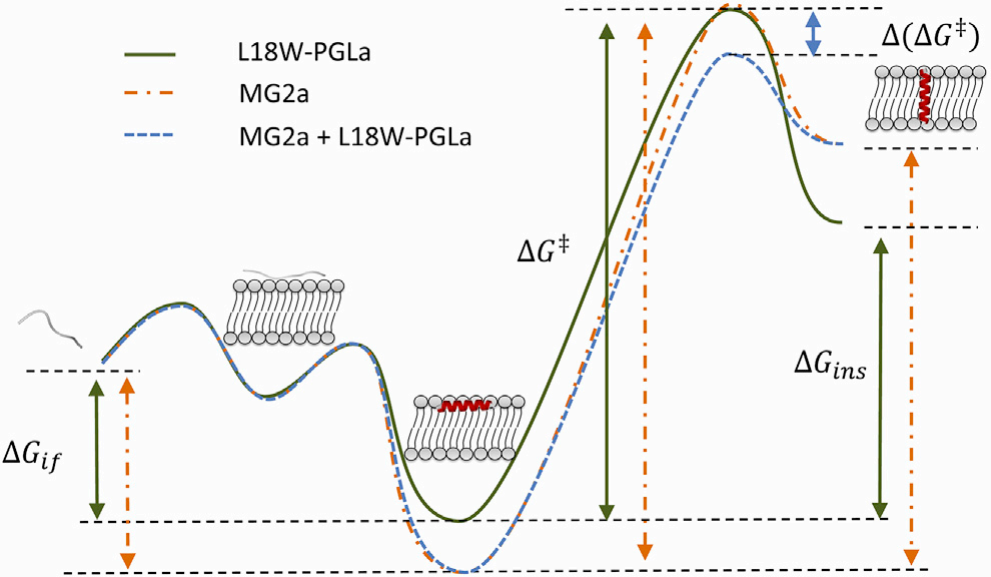
Schematic free-energy landscape with different states during the peptide-insertion process into a lipid bilayer, including adsorption to and folding at the membrane surface $\Delta G_{if}$, as well as insertion into the hydrophobic core $\Delta G_{ins}$, for which the peptide has to overcome an activation barrier $\Delta G^{‡}$. Depending on the energy difference between the transition state (‡) and the peptide’s interfacial state, $\Delta G^{‡}$ can vary significantly. $\Delta G_{if}$ is assumed to be smaller for L18W-PGLa than for MG2a. Synergistic interactions between the peptides may lower the barrier by $\Delta \left(\Delta G^{‡}\right)$. To see this figure in color, go online.

To correlate these considerations with our leakage data, it is further necessary to consider $\Delta G^{‡}$ as introduced above. Inspired by (44), we can draw a hypothetical energy path for AMP insertion and synergism (Fig. 7). In particular, the difference in $\Delta G_{if}$ between L18W-PGLa and MG2a (Table 2) should lead to a higher $\Delta G^{‡}$ for MG2a in bilayers of equal lipid composition, which would explain why MG2a is always less active than L18W-PGLa. In the case of synergistic interaction between L18W-PGLa and MG2a, the activation barrier also appears to be lowered. We speculate in particular that the perturbation of the lipid bilayer by L18W-PGLa helps to lower $\Delta G^{‡}$ for MG2a, which, because of its higher mean hydrophobic moment $\mu_{H}\;$ (Table 2), i.e., amphipathicity, will cause a stronger impairment of the bilayer’s permeability barrier because of a larger mismatch with the polarity profile of the lipid bilayer. This agrees with a previous analysis of properties of *α*-helical antimicrobial peptides showing that their efficacy is dominated by their overall amphipathicity (46). Note that previous solid-state NMR measurements did not report on PGLa-mediated insertion of MG2 (18, 19). However, detection of such states could have been impeded by a limited degree of sample hydration, which has been shown previously to significantly increase threshold concentrations for peptide insertion into lipid bilayers (see, e.g., (47)).

### Synergism is not necessarily coupled to L18W-PGLa/MG2a pair formation

Motivated by a maximum of antimicrobial activity at equimolar peptide ratio, a number of studies claim that synergism of magainins is due to a pairwise interaction of the two AMPs (formation of physical heterodimers) in the presence of the lipid bilayer (13, 15, 16, 33). Further, molecular dynamics simulations (48) and mutation studies (31) suggest that close contact interactions between glycine and alanine residues on PGLa support the formation of an antiparallel (physical) homodimer. Indeed, chemically cross-linked L18W-PGLa homodimers are almost as active as L18W-PGLa-MG2a heterodimers in POPE/POPG (Fig. 4 *A*). These results are in good agreement with several previous studies demonstrating that peptide aggregates (dimers) are in general—due to their larger size—significantly more perturbing bilayers than their monomers (see, e.g., (49, 50, 51, 52)). Nishida et al. (16) argued that association of L18W-PGLa and MG2a is concentration-dependent and that cysteine-linked L18W-PGLa-MG2a mimic high peptide concentrations. However, even at our highest peptide concentration, which is ∼5 times larger than that applied in (16), the heterodimers are still 2–3 times more effective than the equimolar L18W-PGLa/MG2a mixture. Considering that peptides diffuse in membranes at a similar rate to lipids (53, 54), it thus occurs that L18W-PGLa/MG2a pairs should have formed rapidly on the time scale of our experiment. Thus, aggregation of L18W-PGLa and MG2a is not a strict prerequisite for developing synergism. In turn, our conclusion is in agreement with (41), who found that carboxyterminal-GGS or -GGA analogs of PGLa and MG2 are more active than PGLa/MG2a mixtures, although they are not able to form cysteine cross-links.

### Correlation of membrane-lytic activities of magainins between plasma membrane mimics and live cells

The role of membrane lipid architecture in AMP selectivity has been extensively discussed before (see, e.g., (29)). One of the most obvious differences in lipid architecture is the absence of charged lipid species in the outer leaflet of mammalian plasma membranes, whereas bacterial plasma membranes contain anionic lipid species. Thus, attractive electrostatic interactions between cationic residues on AMP and anionic lipids are an essential first step of discrimination that has been already recognized for several years (for review, see, e.g., (6)). However, the effects of membrane elasticity in particular at the polar/apolar interface are of similar importance to consider. This is convincingly demonstrated by comparing the synergistic peptide activities found for POPE/POPG with the peptide activity on *E. coli*. For POPC/POPG, in turn, we found no such agreement. Moreover, the increased activity of L18W-PGLa-MG2a in POPC/Chol mixtures correlates well with their increased hemolysis of erythrocytes (16). We note, however, that other aspects, such as differences in L18W-PGLa and MG2a activities or the almost-equal killing efficacy of equimolar L18W-PGLa/MG2a mixtures and L18W-PGLa-MG2a heterodimers, are not mirrored in our leakage experiments on POPE/POPG (3:1 mol/mol) LUVs*.* This indicates limitations of the currently applied lipid-only model systems, which are unable to capture the full complexity of the *E. coli* cell envelope. We are currently developing more complex membrane mimetic systems.

## Conclusion

We have provided experimental evidence for the significance of combining a negatively charged membrane surface with a tightly packed polar/apolar interface for synergistic interactions of L18W-PGLa and MG2a with lipid membranes. Tight packing of this interface is achieved here by POPE, which has a significant negative intrinsic curvature. Cardiolipin, which is also present in cytoplasmic membranes of Gram-negative bacteria (20) and which has negative *J*_*0*_ in the presence of divalent ions (22), may further add to the interfacial packing strain. If bilayers have negligible overall intrinsic curvature, L18W-PGLa and MG2a permeabilize the membrane much more easily and do not develop synergism. This effect is expected to be even more pronounced for lipids with positive intrinsic curvature, which agrees well with previous observations (18, 19). Consequently, “softening up” the polar/apolar interface by a concerted action of MG2a and L18W-PGLa appears to be the underlying mechanism of synergism for the two peptides. We speculate that L18W-PGLa is the “helper molecule” that preconditions the bilayer and thereby allows MG2a, which, due to its more pronounced amphipathicity, is less compatible with the distribution of polar and nonpolar moieties in lipid membranes, to increase its activity. In addition, synergism also requires electrostatic interactions between the peptides and membrane lipids to anchor the AMPs more strongly within the membrane interface. Importantly, however, pairwise interactions of L18W-PGLa and MG2a are not strictly required for this scenario, which is supported by our experiments on homo- and heterodimers of the two peptides and which agrees with a recent report (41). Additional effects, such as peptide-induced lipid phase separation, may further influence this mechanism. Corresponding experiments will be performed in our laboratory in the near future. The correlation of synergistic activity of L18W-PGLa and MG2a in *E. coli* with dye-release experiments in POPE/POPG LUVs but the inability to reproduce other features of AMP activity in bacteria strongly supports a proper choice of the lipid mimetic system for unraveling the biophysics of AMP-membrane interactions.

## Author Contributions

R.L. performed research, analyzed data, and wrote the article. M.P. performed research and analyzed data. I.K. performed MC simulations. I.S. performed experiments on *E. coli*. D.E. performed research. R.V., G.P., and K.L. designed research and wrote the article.

## Supporting Citations

References (55, 56, 57, 58, 59, 60, 61, 62, 63, 64, 65) appear in the Supporting Material.

## Supporting Material

Document S1. Supporting Materials and Methods, Figs. S1–S5, and Tables S1 and S2

Document S2. Article plus Supporting Material
