# Supplementary material for: Synergism of Antimicrobial Frog Peptides Couples to Membrane Intrinsic Curvature Strain
Source: Biophys J. 2018 Apr 25;114(8):1945–54. doi: 10.1016/j.bpj.2018.03.006 (PMC5937145; doi:10.1016/j.bpj.2018.03.006)
Supplement: Document S1. Supporting Materials and Methods, Figs. S1–S5, and Tables S1 and S2 [file mmc1.pdf]

Biophysical Journal, Volume *114*

Supplemental Information

Synergism of Antimicrobial Frog Peptides Couples to Membrane Intrinsic Curvature Strain

Regina Leber, Michael Pachler, Ivo Kabelka, Irene Svoboda, Daniel Enkoller, Robert Vácha, Karl Lohner, and Georg Pabst

Supplementary Information

Synergism of Antimicrobial Frog Peptides Couples to Membrane Intrinsic Curvature Strain

Regina Leber, Michael Pachler, Ivo Kabelka, Irene Svoboda, Daniel Enkoller, Robert Vácha, Karl Lohner, and Georg Pabst

PREPARATION OF LIPOSOMES

Lipid films (20 mg total lipid) for dye-release experiments were prepared by mixing the appropriate amounts of dissolved phospholipids before evaporation of solvents under a stream of nitrogen and stored in vacuum overnight. After addition of Hepes buffer containing the fluorophor/quencher ANTS/DPX (10 mM Hepes, 68 mM NaCl, 12.5 mM ANTS, 45 mM DPX, pH 7.4), formation of lipid vesicles was achieved by equilibration at 15°C above the lipid's melting temperature and intermittent vigorous vortex mixing. PE/PG-mixtures were additionally homogenized by six freeze/thaw-cycles. Large unilamellar vesicles (LUVs) were obtained by 25 cycles of extrusion of the hydrated liposomes through a polycarbonate filter (Millipore-Isopore™, Merck, Vienna) of 0.1 µm pore size above the lipid's melting temperature. Non-encapsulated fluorophore and quencher were removed by size-exclusion chromatography using SephadexTM G-75 (Sigma-Aldrich, Vienna) gel as described earlier (1). LUVs were eluted with Hepes buffer (10 mM Hepes, 140 mM NaCl, pH 7.4). Liposomal lipids were quantified by the determination of lipid phosphorus according the method described by (2). The final LUV concentration for dye-release experiments was adjusted to 50 µM in Hepes buffer (10 mM Hepes, 140 mM NaCl, pH 7.4).

PREPARATION AND ANALYSIS OF INVERTED HEXAGONAL PHASES BY SAXS

Samples for X-ray analysis were prepared by rapid solvent exchange (RSE) (3) using an adapted method that allows control of temperature (4). Here we focus in particular on the intrinsic curvatures of POPG and lyso-PE, whose values have to the best of our knowledge not been reported before. Both lipids do not form H_{II} phases and were therefore mixed with DOPE at various amounts as detailed above. In brief, appropriate amounts of lipid stock solution were pipetted into test tubes containing NaPi buffer (20 mM Na-phosphate, 130 mM NaCl, pH 7.4) and which were pre-equilibrated at a given temperature. The test tubes were quickly mounted onto the RSE apparatus, setting the argon flow to 60 ml/min and the vortex speed to 1000-1200 rpm. Rapid evaporation was initiated by opening the valve to a vacuum

pump set to a pressure in between the vapor pressure of solvent and water leading to the formation of lipid aggregates. Sample preparation was terminated after six minutes. Details of RSE settings are listed in supplementary Table S1. Control experiments using ^{1}H NMR showed no contamination of the samples with organic solvent.

Table S1: Settings used for the rapid solvent exchange.

Lipids	Temperature [°C]	Pressure [mbar]	Vortex speed [U/min]
DOPE	45	200	1200
POPG/DOPE	60	300	1000
lyso-PE/DOPE	70	900	1000
POPE	55	360	1000

All samples contained 12 wt% tricosene, which is needed to reduce the packing frustration of the inverted hexagonal phase (H_{II}) (see, e.g., (5)). This effect decreases the lamellar-to-H_{II} phase transition of POPE close to room temperature (6). POPG and lyso-PE do not form H_{II} phases. Therefore they were mixed with DOPE at various concentrations using a previously reported protocol (6). Samples containing DOPE and POPG were measured immediately after preparation, while lyso-PE containing samples were equilibrated for three days at room temperature in argon atmosphere to avoid lipid oxidation.

SAXS measurements were performed using a SAXSpace compact camera from Anton Paar (Austria), equipped with a Pilatus 100K-S detector system (Dectris, Switzerland) and a Genix 3D microfocus X-ray generator from Xenocs (Grenoble, France) [beam-size (Cu-Kα): ~ 300 µm (diameter)]. A scattering vector range of q = 0.6 nm^{-1} o 10.9 nm^{-1} was covered by setting the sample-to-detector distance to 308 mm. Temperature control was provided by a Peltier unit to within ± 0.1 °C. All samples were transferred into a paste cell holder (Anton Paar, Austria) and equilibrated for 10 minutes at 35°C before exposing to X-rays for one hour. Data reduction was performed with SAXStreat (Anton Paar, Austria). Note that temperature dependencies of intrinsic curvatures are in the range of $\frac{\Delta J_0}{T} = -1 \times 10^{-3}$ nm^{-1}/K^{-1} to -3×10^{-3} nm^{-1}/K^{-1} (6). Temperature corrections to adjust the here reported J_0's to 37°C used for vesicle leakage assays are consequently within experimental error.

SAXS data analysis was performed according to Kollmitzer et al. (6). In brief, intensities and peak positions of H_{II} phase patterns $I_{h,k}$ were used to construct electron density maps

$$\rho(\vec{r}) = \sum_{h,k} \alpha_{h,k} \sqrt{\frac{I_{h,k} q_{h,k}^2}{m_{h,k}}} \cos\left(2\pi \overrightarrow{q_{h,k}}\, \vec{r}\right), \qquad \text{(S1)}$$

where h, k are the Miller indices, $m_{h,k}$ is the multiplicity of equivalent diffraction planes and $\alpha_{h,k}$ is the phase. Subsequently, electron density maps were radially integrated over azimuthal angles between 0° and 60° to yield one-dimensional electron density profiles. The maximum of these profiles was fitted with a Gaussian to determine the position of the lipid headgroup R_p. The intrinsic curvature $J_0 = -1/R_0$ is then determined by finding the position of the neutral plane $R_0 = R_p + d_{H1}$ (7), which is assumed to be located at the glycerol backbone (6). Specifically, we used $d_{H1} = 0.44$ nm as estimate for the distance between the lipid heads and the backbone (6).

The intrinsic curvature of non H_{II}-phase forming lipids was estimated assuming linear additivity using

$$J_0 = \frac{J_0^{mix} - (1-\chi) J_0^{DOPE}}{\chi}, \qquad \text{(S2)}$$

where χ is the mole fraction of the respective 'guest' lipid in DOPE, which is used as a H_{II}-phase forming template ('host'). To increase the reliability of the J_0 estimates for guest lipids several guest/host mixtures were measured and interpolated, ensuring full miscibility of the guest lipids within the host structure (Fig. S1) and (6).

Figure S1 shows the SAXS patterns of 9:1 (molar ratio) lipid mixtures used to calculate J_0. The intrinsic curvatures of both DOPE mixtures increased with concentration of the guest lipid (Fig. S1, insert), albeit stronger for lyso-PE, as can be expected from its cone-like molecular shape, due to the difference of projected lateral areas of the PE head and the single hydrocarbon chain (8). The resulting J_0's for all lipids relevant for this study are listed in Tab. S2. In agreement with the qualitative assessment based on the molecular shape we found a significant positive J_0 for lyso-PE, while J_0 of POPG was, within experimental uncertainty, essentially zero. In order to test the effect of buffer on intrinsic lipid curvatures we also measured POPE, which readily forms in the presence of 12 wt.% tricosene a H_{II} phase at 35°C (Fig. S1), see also (6). The derived J_0 = -0.35 nm^{-1} is slightly more negative than our previously reported value. We attribute this to the effect of buffer ions on PE headgroup hydration, which is further supported by a similar decrease of J_0 for DOPE (-0.45 nm^{-1}) as compared to the value found in pure water (6). For the present study such subtleties are, however, of less significance. Here we are interested in estimates for net intrinsic curvatures

of lipid mixtures, which were calculated by their molecular averages upon assuming linear additivity

$$J_0^{mix} = \sum_i \chi_i J_0^{(i)}. \qquad (3)$$

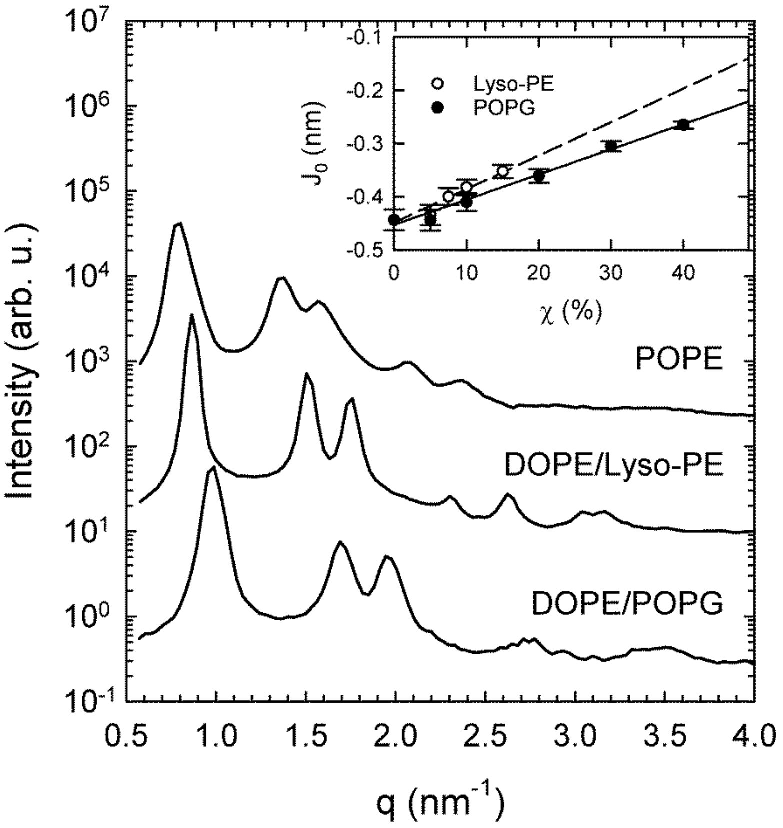

Figure S1: SAXS patterns of DOPE/POPG (9:1 mol/mol), DOPE/lyso-PE (9:1 mol/mol) and POPE at 35°C. All samples contained 12 wt% tricosene. Data are offset vertically for clarity of presentation. The insert shows the change of intrinsic curvatures as a function of POPG and lyso-PE concentration in DOPE, which is used to estimate their J_0's.

Table S2: Intrinsic curvatures of presently studied lipids at 35°C.

**Lipid**	J_0 **[nm^{-1}]**
lyso-PE	0.180 ± 0.123
POPG	0.020 ± 0.030
POPE	-0.350 ± 0.007
POPC*	-0.022 ± 0.010
Chol*	-0.494 ± 0.013

* Data taken from ref. (6)

FLUORESCENCE SPECTROSCOPY - LEAKAGE ASSAY

Leakage of the aqueous content of ANTS/DPX-loaded LUVs upon incubation with peptides was determined according to (9) and as detailed previously (1). Briefly, 2 ml of LUV suspension was filled into quartz cuvettes and equilibrated at 37°C for 5 minutes. Incremental amounts of peptide solutions prepared in Hepes buffer were added to the LUV suspension with µ-pipettes and mixed using a magnetic stirrer to achieve final peptide concentrations ranging from 0.016 - 1 µM, corresponding to peptide-to-lipid molar ratios from 1:3200 to 1:50. Fluorescence emission was recorded 5 minutes before and after the addition of peptides on a Cary Eclipse Fluorescence Spectrophotometer (Varian Inc., now Agilent Technologies, California, US) using an excitation wavelength of λ_{ex} = 360 nm and an emission wavelength λ_{em} = 530 nm. The slit widths for excitation and emission monochromators were set to 10 nm. Percentage of leakage was calculated from the fraction of the leakage (10)

$$I_F = \frac{(F - F_0)}{(F_{max} - F_0)}, \qquad \text{(S4)}$$

where F is the measured fluorescence, F_0 is the initial fluorescence without peptide, and F_{max} is the fluorescence corresponding to 100% leakage gained by addition of 1% Triton X-100.

SODIUM DODECYL SULFATE POLYACRYLAMIDE GEL ELECTROPHORESIS (SDS-PAGE)

In order to verify the dimeric or monomeric state of peptides, 2 µg of peptides were dissolved in loading buffer without DTT and separated by a one-dimensional SDS-PAGE using 5 % stacking and 18 % separating gels according to the method of Laemmli (11). Peptides were stained for 30 min with Coomassie brilliant blue after electrophoretic separation (11). An ultra-low range molecular weight marker was used to estimate the size of peptides after separation via SDS-PAGE. Experiments show that L18W-PGL-GGCa and MG2-GGCa form homodimers in buffer solution (pH ~ 7) (Fig. S2), in agreement with (12). Application of DTT leads to a drop of peptide activity to levels observed for single peptides, consistent with monomer formation (Fig. S3).

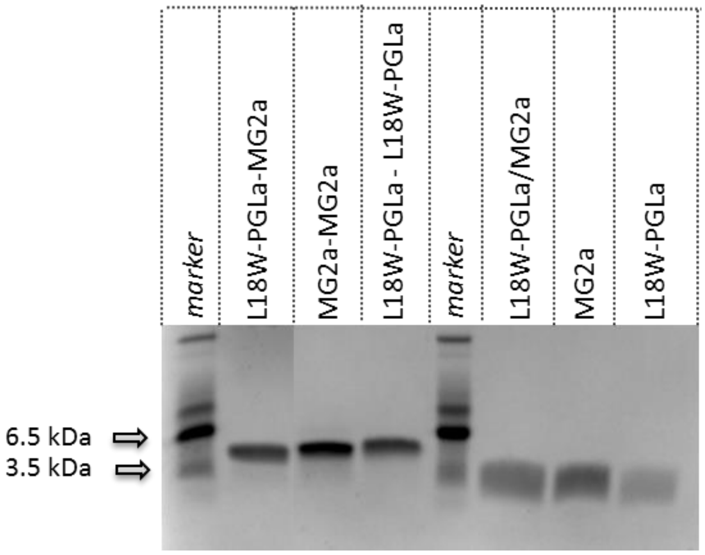

Figure S2: SDS-PAGE verifying dimeric and monomeric forms of magainins

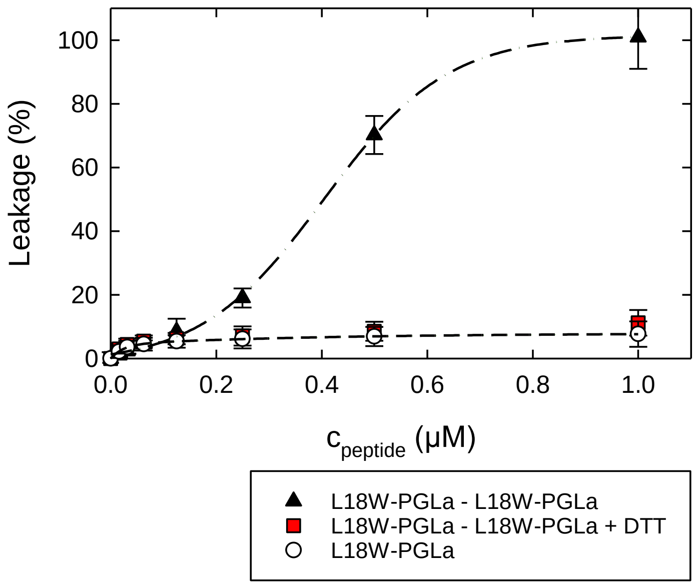

Figure S3: Effect of DTT addition to L18W-PGLa homodimers on leakage of POPE/POPG 3:1 mol/mol LUVs. For comparison, leakage induced by L18W-PGLa is shown as well (see also Fig. 2 *B*). The lipid concentration was 50 µM. Lines are guides to the eye.

MC SIMULATIONS

Monte Carlo simulations were performed using the Metropolis scheme and computationally efficient implicit solvent coarse-grained models. Sizes and distances of the models were calculated in reduced units, which (based on real particle dimensions) roughly translate to nm. The studied systems were composed of 200 lipid molecules and a single peptide. Lipids were

described by a three-bead model developed by Cooke and Deserno (13), using one bead for the head group of diameter d_h and two beads for tails each of diameter d_t (Fig. S4 *A*).

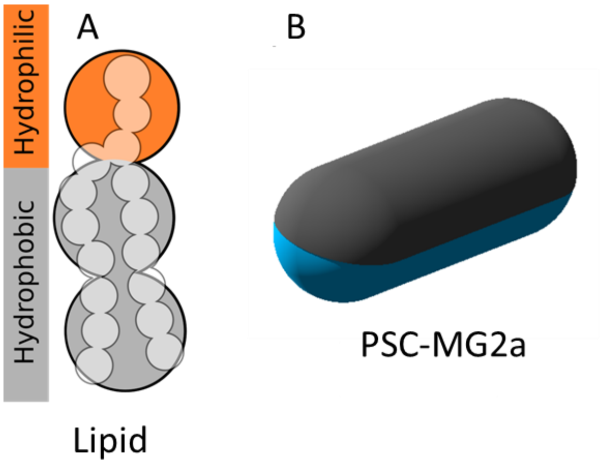

Figure S4: Schematic representations of the employed models. Panel A: Three-bead lipid model with lipid mapping (shown as silhouette). Panel B: PSC model of MG2a. Hydrophobic patches/regions are represented in grey, while orange and blue depict hydrophilic parts of lipid and peptide, respectively.

Three lipids with different intrinsic curvatures were simulated by variation of headgroup size (Fig. S5). The size of head bead affects J_0. Assuming cylindrical geometry of lipids one can calculate the intrinsic curvature by (14)

$$J_0 = \frac{2}{l}\left(1 - \frac{V}{A_h l}\right), \quad \text{(S5)}$$

where l is the length of the hydrophobic tails, A_h is the effective headgroup area and V is the hydrocarbon chain volume. However, there are more possibilities to calculate the l and $V/(A_h l)$. The first possibility is based on the geometry of individual molecules with $A_h = \pi d_h^2/4$, $V = \pi d_t^2 l/4$, and $l = 2d_t$. For $d_h = 0.85$ nm, 0.95 nm and 1.05 nm, this results for $d_t = 1$ nm in $J_0 = -0.38$ nm^{-1}, -0.11 nm^{-1}, and $+0.09$ nm^{-1}, respectively. However, this considers only excluded volume interactions between lipid molecules in ideal configuration. We therefore adhere to the second possibility, which derives J_0 from MC simulations and consequently include also contributions from temperature and the lipid parametrization (bonds, bending, and tail attraction). In this case the length of hydrophobic tails is calculated from the average distance of headgroup beads from the bilayer center, i.e. $d_l = 2.5$ nm. The headgroup areas are derived from the simulation box size of the equilibrated membrane at zero tension, which yields $A_h = 1.025$ nm^2, 1.145 nm^2 and 1.215 nm^2 with increasing d_h. Assuming that $A_h \propto d_h^2$ we can interpolate the headgroup area to perfectly cylindrical lipids (i.e., $d_h = d_t$ and $J_0 = 0$), yielding $A_h^0 = 1.176$ nm^2. Consequently Eq. (S5) is modified to

$$J_0 = \frac{2}{l}\left(1 - \frac{A_h^0}{A_h}\right), \tag{S6}$$

leading to $J_0 = -0.12$ nm^{-1}, -0.02 nm^{-1}, and $+0.03$ nm^{-1} for $d_h = 0.85$ nm, 0.95 nm and 1.05 nm, respectively. The lipid with the largest headgroup leads to $J_0 > 0$.

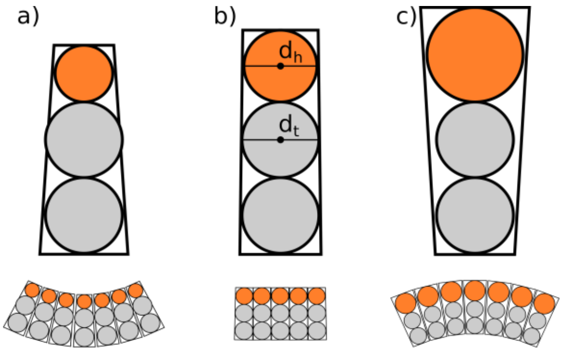

Figure S5: Schematic representation of lipid shape and induced curvature. Diameters of lipid heads and tails are denoted as d_h and d_t, respectively, with d_t = 1 nm. Conical lipids a) and c) had d_h = 0.85 and 1.05 nm, respectively. For cylindrical lipids b) d_h = 0.95 nm. The corresponding intrinsic curvatures are a) J_0 = -0.12 nm^{-1}, b) J_0 = -0.02 nm^{-1}, and c) J_0 = +0.03 nm^{-1}.The induced curvature is shown below each lipid. Differences in headgroup sizes are magnified for clarity.

The peptide was modelled by a patchy spherocylinder (PSC) (15), setting the peptide length to 4 nm and its diameter to 1 nm to roughly match the size and polarity of MG2a, i.e. half of the peptide surface was hydrophobic (Fig. S4 *B*). At either side of the patch, there is a 5° switching range to linearly scale the interaction potential to zero. The remaining surface is purely repulsive and thus considered to be hydrophilic. For more details on the model, see (15).

To mimic the hydrophobic effect, effective tail-tail interactions were used to drive membrane self-assembly leading to an effective attractive potential with a $\cos^2$ profile. Further, the same potential was used to describe interactions between lipid tails and the peptide's hydrophobic patch. Repulsive interactions were modeled by Weeks-Chandler-Anderson (shifted and truncated Lennard-Jones) potential. The PSC-MG2a switching range between hydrophobic and hydrophilic surfaces was set to 1.0 nm. As described in the original article, the switching range of lipids was extended to 1.6 nm to keep the membrane in the fluid phase (13). The depth of the attractive potential well was -1 kT (k is the Boltzmann constant and T is the temperature) for lipid-tail particles and -1 kT per unit length of PSC line segment.

The membrane bilayer was assembled in the XY-plane of a rectangular box. The initial box size was about $11 \times 11 \times 50$ nm. Periodic boundary conditions were applied in all three

dimensions, forming "infinite" membrane plane. Simulations were performed under constant temperature and number of particles, while keeping zero lateral tension in the XY-plane. We used standard MC trial moves to move the peptide/lipid beads/whole lipids and to modify the box size (16). All simulations were carried out using our in-house software (freely available github.com/robertvacha/SC).

Free energy calculations for peptide translocation were performed using the Wang-Landau method (17). The free energy surface was considered converged once the modification factor was below 10^{-7}. Subsequently, simulations maintaining detailed balance of peptide translocation were performed and the obtained free energy surface was used as an external bias. The obtained histogram was then used to further improve the previously calculated free energy surface. The error of the free energy profiles is about 1 kT.

For proper sampling of peptide insertion, two collective variables (CVs) were used. First CV is the distance between peptide and the membrane center of mass. The free energy profile is symmetrical around the membrane center and asymmetry shows the calculation error. Second CV is the peptide orientation with respect to the membrane normal. The orientation is expressed as the cosine of the angle. Change of orientation from perpendicular to parallel (with respect to the membrane normal) is considered. For clarity, the second collective variable is averaged out and only the dependence on peptide position is shown.

IN VITRO ASSAY FOR AMP ACTIVITY

To study the effect of magainins on metabolically active bacteria, we assessed the antimicrobial activity of the peptides by determining the MIC values according to Clinical and Laboratory Standards Institute (CLSI, formerly National Committee for Clinical Laboratory Standards) guidelines.

Inhibition of bacterial growth by frog peptides was determined using an automated turbidimetric-based system (Bioscreen C, Labsystem, Helsinki, Finland), which measures absorbance of cell cultures in 100-well plates at regular time intervals (λ = 580 nm). Assays were performed in Mueller-Hinton (MH) broth without Ca^{2+} (18) using Bioscreen polystyrene honeycomb 100-well plates. Briefly, serial dilutions of the AMPs or control antibiotics (gentamicin and ampicillin B) were made in MH broth and dispensed with a multi-pipette into

polystyrene honeycomb 100-well plates. *E. coli* K12 cells grown for 18–20 h on agar plates were suspended in saline adjusting the turbidity to an optical density at λ=565 nm (OD_{565}) of 0.5 (~1 × 10^8 cells/mL) using a DEN-1B densitometer (Grant Instruments, Cambridge, UK). A fifty-fold dilution of this *E. coli* suspension prepared in MH broth was mixed with 50 µL (1:1 volume ratio) of a given peptide solution by three times up and down pipetting. Resulting cell suspensions were grown at 37°C with continuous, low amplitude shaking. The turbidity was measured in intervals of 5 minutes (filter bandwidth: λ= 420 – 580 nm). MIC was considered as the minimum concentration of peptide that inhibited any increment of turbidity after 48 h.

REFERENCES

1. Zweytick, D., G. Deutsch, J. Andrä, S. E. Blondelle, E. Vollmer, R. Jerala, and K. Lohner. 2011. Studies on lactoferricin-derived Escherichia coli membrane-active peptides reveal differences in the mechanism of N-acylated versus nonacylated peptides. J Biol Chem 286:21266–21276.
2. Broekhuyse, R. M. 1968. Phospholipids in tissues of the eye. I. Isolation, characterization and quantitative analysis by two-dimensional thin-layer chromatography of diacyl and vinyl-ether phospholipids. Biochim Biophys Acta 152:307–315.
3. Buboltz, J. T., and G. W. Feigenson. 1999. A novel strategy for the preparation of liposomes: rapid solvent exchange. Biochim Biophys Acta 1417:232–245.
4. Rieder, A. A., D. Koller, K. Lohner, and G. Pabst. 2015. Optimizing rapid solvent exchange preparation of multilamellar vesicles. Chemistry and physics of lipids 186:39–44.
5. Alley, S. H., O. Ces, M. Barahona, and R. H. Templer. 2008. X-ray diffraction measurement of the monolayer spontaneous curvature of dioleoylphosphatidylglycerol. Chemistry and physics of lipids 154:64–67.
6. Kollmitzer, B., P. Heftberger, M. Rappolt, and G. Pabst. 2013. Monolayer spontaneous curvature of raft-forming membrane lipids. Soft Matter 9:10877.
7. Leikin, S., M. M. Kozlov, N. L. Fuller, and R. P. Rand. 1996. Measured effects of diacylglycerol on structural and elastic properties of phospholipid membranes. Biophys J 71:2623–2632.
8. Israelachvili, J. N. 2011. Intermolecular and surface forces, 3rd ed. Academic Press, Burlington, MA.
9. Ellens, H., J. Bentz, and F. C. Szoka. 1985. H+- and Ca2+-induced fusion and destabilization of liposomes. Biochemistry 24:3099–3106.
10. Ladokhin, A. S., W. C. Wimley, and S. H. White. 1995. Leakage of membrane vesicle contents: Determination of mechanism using fluorescence requenching. Biophys J 69:1964–1971.

11. Laemmli, U. K. 1970. Cleavage of structural proteins during the assembly of the head of bacteriophage T4. Nature 227:680–685.

12. Hara, T., Y. Mitani, K. Tanaka, N. Uematsu, A. Takakura, T. Tachi, H. Kodama, M. Kondo, H. Mori, A. Otaka, F. Nobutaka, and K. Matsuzaki. 2001. Heterodimer formation between the antimicrobial peptides magainin 2 and PGLa in lipid bilayers: a cross-linking study. Biochemistry 40:12395–12399.

13. Cooke, I. R., K. Kremer, and M. Deserno. 2005. Tunable generic model for fluid bilayer membranes. Phys Rev E 72:11506.

14. Marsh, D. 1996. Intrinsic curvature in normal and inverted lipid structures and in membranes. Biophys J 70:2248–2255.

15. Vácha, R., and D. Frenkel. 2011. Relation between molecular shape and the morphology of self-assembling aggregates: a simulation study. Biophys J 101:1432–1439.

16. Frenkel, D., and B. Smit. 2002. Understanding Molecular Simulation. Academic Press, San Diego, CA.

17. Wang, F., and D. P. Landau. 2001. Efficient, multiple-range random walk algorithm to calculate the density of states. Phys Rev Lett 86:2050–2053.

18. Mueller, J. H., and J. Hinton. 1941. A protein-free medium for primary isolation of the gonococcus and meningococcus. Proc Soc Exp Biol Med 48:330–333.
