# Supplementary material for: Synergism of Antimicrobial Frog Peptides Couples to Membrane Intrinsic Curvature Strain
Source: Biophys J. 2018 Apr 25;114(8):1945–54. doi: 10.1016/j.bpj.2018.03.006 (PMC5937145; doi:10.1016/j.bpj.2018.03.006)
Supplement: Document S2. Article plus Supporting Material [file mmc2.pdf]

Biophysical Journal
Article

Biophysical Society

Synergism of Antimicrobial Frog Peptides Couples to Membrane Intrinsic Curvature Strain

Regina Leber,[1,2] Michael Pachler,[1,2] Ivo Kabelka,[3,4] Irene Svoboda,[1,2] Daniel Enkoller,[5] Robert Vácha,[3,4] Karl Lohner,[1,2] and Georg Pabst[1,2,*]

[1]Institute of Molecular Biosciences, Biophysics Division, University of Graz, NAWI Graz, Graz, Austria; [2]BioTechMed Graz, Graz, Austria; [3]Central European Institute of Technology, Brno, Czech Republic; [4]Faculty of Science, Masaryk University, Brno, Czech Republic; and [5]Fresenius Kabi Austria GmbH, Graz, Austria

ABSTRACT Mixtures of the frog peptides magainin 2 and PGLa are well-known for their pronounced synergistic killing of Gram-negative bacteria. We aimed to gain insight into the underlying biophysical mechanism by interrogating the permeabilizing efficacies of the peptides as a function of stored membrane curvature strain. For Gram-negative bacterial-inner-membrane mimics, synergism was only observed when the anionic bilayers exhibited significant negative intrinsic curvatures imposed by monounsaturated phosphatidylethanolamine. In contrast, the peptides and their mixtures did not exhibit significant activities in charge-neutral mammalian mimics, including those with negative curvature, which is consistent with the requirement of charge-mediated peptide binding to the membrane. Our experimental findings are supported by computer simulations showing a significant decrease of the peptide-insertion free energy in membranes upon shifting intrinsic curvatures toward more positive values. The physiological relevance of our model studies is corroborated by a remarkable agreement with the peptide's synergistic activity in *Escherichia coli*. We propose that synergism is related to a lowering of a membrane-curvature-strain-mediated free-energy barrier by PGLa that assists membrane insertion of magainin 2, and not by strict pairwise interactions of the two peptides as suggested previously.

INTRODUCTION

Antimicrobial peptides (AMPs) are highly effective components of the innate immune system of most living organisms. AMPs respond to invading pathogens and are optimized to kill bacteria either by direct interaction or by immunomodulatory activities (1–3). Ever since the discovery of AMPs in prokaryotes in the 1930s and a few decades later in eukaryotes, research efforts devoted to the molecular mechanisms of their direct antimicrobial activities have revealed several modes of interaction (for review, see, e.g., (4,5)). A general consensus has been reached in recognizing that the positive charge of the peptide is essential for initial binding to the anionic bacterial membrane surface, a factor which allows discrimination between bacterial and host cell membranes, whereas hydrophobicity is needed for insertion into and disruption of the target membrane (see, e.g., (6,7)). Magainin 2 (MG2) and PGLa, two members of the magainin family isolated from the skin of the African clawed frog *Xenopus laevis* (8,9), are among the best-studied AMPs. They exhibit a broad spectrum of activity against microorganisms with minimal inhibitory concentration (MIC) values ranging from 5 to >100 μg/mL (10). Of particular interest is their synergistic activity, which has been observed in bacteria but also in lipid-only model membranes (11–16). Notably, MG2/PGLa synergism is most pronounced for Gram-negative strains such as *Escherichia coli* (13).

Unraveling the molecular origin of this synergistic activity has not been without disparity. Early biophysical studies on lipid-only systems suggested enhanced membrane-pore-formation capabilities of equimolar peptide mixtures (13–15). Indeed, a stable transmembrane orientation of PGLa/MG2 dimers, as reported from solid-state NMR measurements, supported this model (17). A few years later, however, Salnikov and Bechinger showed that the topology of PGLa and MG2 (i.e., their orientation with respect to the bilayer surface), and in particular that of MG2, depends on the hydrophobic thickness of the lipid bilayer (18). The important role of membrane physical parameters was further emphasized by Strandberg et al. (19) by correlating the topology of both peptides with the intrinsic curvature of lipids. In particular, these authors suggested that the

Submitted January 9, 2018, and accepted for publication March 12, 2018.
*Correspondence: georg.pabst@uni-graz.at
Editor: Kalina Hristova.
https://doi.org/10.1016/j.bpj.2018.03.006
© 2018 Biophysical Society.
This is an open access article under the CC BY license (http://creativecommons.org/licenses/by/4.0/).

synergistic insertion of MG2 and PGLa into membranes couples to a positive intrinsic curvature.

Intriguingly, however, cytoplasmic membranes of Gram-negative bacteria are enriched in lipids with negative intrinsic curvature, such as phosphatidylethanolamine (PE) (20). This prompted us to correlate membrane-permeabilizing activities of magainin monomers and dimers in different lipid-only model systems with various intrinsic curvatures J_0 determined from small-angle x-ray scattering (SAXS). In particular, we studied the activity of L18W-PGLa and MG2a, as well as their analogs containing GGC linkers, which were used to form the L18W-PGLa-MG2a hybrid peptide (Fig. 1). The choice of L18W-PGLa is motivated to facilitate better comparison to a previous study on magainin heterodimers (15). Note that the dye release from lipid vesicles induced by L18W-PGLa is almost identical to that induced by native PGLa (13). Thus, our results can be generalized to PGLa in our comparison to other reports. Likewise, MG2a is discussed synonymously with MG2.

The choice of lipid-only mimics is delicate. In addition to the PE enrichment, cytoplasmic membranes of Gram-negative bacteria also contain anionic phospholipids such as phosphatidylglycerol (PG) and cardiolipin (CL). The relative abundance of anionic lipids strongly depends on the strain (20) and growth conditions (21). For instance, the inner membrane of *Pseudomonas cepacia* contains 82% PE and 18% PG (wt% of total phospholipid), whereas *E. coli* also contains ~10 wt% CL (20). The focus of the present work is on the role of intrinsic curvature strain in magainin synergism. Because the sign of J_0 of CL strongly depends on the ionic composition of the aqueous buffer (22), we decided to mimic the inner membrane of Gram-negative bacteria to first order with a binary lipid mixture of POPE/POPG 3:1 mol/mol to avoid any ambiguities resulting from this issue. Additionally, phosphatidylcholine (PC)/cholesterol (Chol) mixtures served as a simple first-order model of human erythrocyte membranes considered as archetypes of mammalian plasma membranes. Again, PC/Chol mimics do not capture the full compositional complexity of such plasma membranes, but serve here to address the role of intrinsic curvature in a system lacking electrostatic interactions between anionic lipids and cationic peptide residues. Our results provide evidence that PGLa/MG2 synergism is tightly coupled to negative lipid intrinsic curvature and to negative surface charge of membranes but does not necessarily require, nor exclude, the formation of PGLa/MG2 pairs. Although electrostatic interactions between AMPs and lipid headgroups are crucial for significantly long retention time (binding) of the peptides to the bilayer surface, lipid intrinsic curvatures contribute to an activation free-energy barrier $\Delta G^{\ddagger}$ for membrane insertion of the peptides. We propose that PGLa, because of its higher affinity for the hydrophobic core of the bilayer, lowers $\Delta G^{\ddagger}$ for MG2 by reducing the curvature-mediated tension at the polar-apolar membrane interface and thereby potentiates the effect of MG2.

L18W-PGLa

GMASKAGAIAGKIAKVAWKAL – NH_2

L18W-PGL-GGCa

GMASKAGAIAGKIAKVAWKAL – GGC – NH_2

MG2a

GIGKFLHSAKKFGKAFVGEIMNS – NH_2

MG2-GGCa

GIGKFLHSAKKFGKAFVGEIMNS – GGC – NH_2

L18W-PGLa–MG2a

GMASKAGAIAGKIAKVAWKAL – GGC – NH_2
|
GIGKFLHSAKKFGKAFVGEIMNS – GGC – NH_2

FIGURE 1 Primary structures of studied magainins.

MATERIALS AND METHODS

Lipids, peptides, and chemicals

Palmitoyl-oleoyl-phosphatidylethanolamine (POPE), dioleoyl-phosphatidylethanolamine (DOPE), palmitoyl-oleoyl-phosphatidylglycerol (POPG), palmitoyl-oleoyl-phosphatidylcholine (POPC), and 1-palmitoyl-2-hydroxy-PE (lyso-PE) were purchased from Avanti Polar Lipids, Alabaster, AL (purity >99%) as powder. Cholesterol, *cis*-9-tricosene, and Triton X-100 were obtained from Sigma-Aldrich, Vienna, Austria. L18W-PGLa and MG2a, as well as their analogs containing GGC linkers and L18W-PGLa-MG2a heterodimers, were obtained in lyophilized form (purity >95%) from PolyPeptide Laboratories (San Diego, CA). ANTS (8-aminonaphthalene-1,3,6-trisulfonic acid, disodium salt) and DPX (p-xylene-bis-pyridinium bromide) were purchased from Molecular Probes (Eugene, OR). Chemicals used for sodium dodecyl sulfate polyacrylamide gel electrophoresis (SDS-PAGE) as well as antibiotics (ampicillin sodium salt, gentamicin sulfate solution), 1,4-dithiothreitol (DTT), and Mueller-Hinton broth were obtained from Carl Roth (Karlsruhe, Germany). The Ultra-Low Range Marker for SDS-PAGE was purchased from Sigma-Aldrich.

Lipid stock solutions for sample preparation were prepared in organic solvent. In particular, lyso-PE was dissolved in chloroform/methanol/water 65:35:8 (v/v/v), whereas all other lipids, including *cis*-9-tricosene, were dissolved in chloroform/methanol (9:1; v/v). Peptide stock solutions were prepared in 0.01% acetic acid, and aliquots of the stock solutions were stored in silanized glass tubes at −20°C until use.

Methods

Large unilamellar vesicles (LUVs) of ~100 nm size were prepared and assayed for AMP-induced fluorescence leakage as described previously

(23). Intrinsic lipid curvatures were determined using a SAXSpace small-angle x-ray camera (Anton Paar, Graz, Austria) applying previously developed procedures (24). Monte Carlo (MC) simulations were performed using the Metropolis scheme and computationally efficient implicit-solvent coarse-grained models. Lipids were described by a three-bead model developed by Cooke and Deserno (25), and the peptide was modeled by a patchy spherocylinder (26). Free-energy calculations for peptide translocation were performed using the Wang-Landau method (27). For details of experimental and simulation procedures, see the Supporting Material.

RESULTS

Intrinsic curvatures of biomimetic membranes

We first determined estimates for J_0 of the studied lipids and their mixtures using SAXS as detailed in (24) (see also Supporting Material and Fig. S1 for x-ray data). Intrinsic curvatures relate to the elastic curvature energy stored in lipid membranes (see, e.g., (28)). The following lipid mixtures are relevant for the present study: POPE/POPG (3:1, mol/mol) as a mimic of the inner membrane of Gram-negative bacteria (29), and POPC/POPG (3:1, mol/mol) as an alternative and frequently used lipid-only model of bacterial membranes (see, e.g., (18,19)). Based on the intrinsic curvatures of the individual lipids (Table S2), we calculate, assuming linear additivity, a significantly negative value for POPE/POPG ($J_0^{mix} = -0.26\ \mathrm{nm}^{-1}$), whereas POPC/POPG is found to have $J_0^{mix} \sim 0$ (Table 1). To investigate the role of intrinsic curvature, we further studied lyso-PE/POPE/POPG (1.6:1.4:1, mol/mol/mol), which has an overall 3:1 PE/PG molar ratio but $J_0^{mix} \sim 0$. Two charge-neutral mimics of mammalian membranes were considered for testing the role of Coulomb interactions in addition to the effect of stored energy strain. Our choice was to use POPC and its 3:1 (mol/mol) mixture with cholesterol, which, because of the negative intrinsic curvature of cholesterol (24), has $J_0^{mix} = -0.14\ \mathrm{nm}^{-1}$, which served as a first-order approximate to mammalian plasma membranes.

Effect of intrinsic curvature and membrane charge

In the next step, we determined ANTS/DPX leakage in LUVs induced by L18W-PGLa, MG2a, and their equimolar mixture to exploit the effect of J_0 on AMP activity. The L18W-PGLa/MG2a mixture is motivated by previous studies (13) suggesting a pairwise interaction of the peptides to achieve synergism. In POPC/POPG ($J_0^{mix} \sim 0$), we found significant peptide activities (Fig. 2 *A*). Specifically, MG2a and L18W-PGLa were similarly permeabilizing vesicles at low concentrations, whereas L18W-PGLa was more effective than MG2a for $c_{peptide} \gtrsim 0.3\ \mu$M. The equimolar L18W-PGLa/MG2a mixture, in turn, was less active than the peptides individually below that concentration, suggesting some antagonizing effect of unknown origin, but one

TABLE 1 Intrinsic Curvatures J_0^{mix} of Membrane Mimetics and Peptide Synergy Σ

Mimic (molar ratio)	J_0^{mix} [nm^{-1}]	Σ
POPE:POPG (3:1)	−0.258 ± 0.013	0.4 ± 0.1
POPC/POPG (3:1)	−0.012 ± 0.015	0.8 ± 0.2
lyso-PE/POPE/POPG (1.6:1.4:1)	−0.044 ± 0.059	1.3 ± 0.3
POPC	−0.022 ± 0.010	2.2 ± 0.4
POPC:Chol (3:1)	−0.140 ± 0.011	1.2 ± 0.3

For definition of Σ, see Eq. 1.

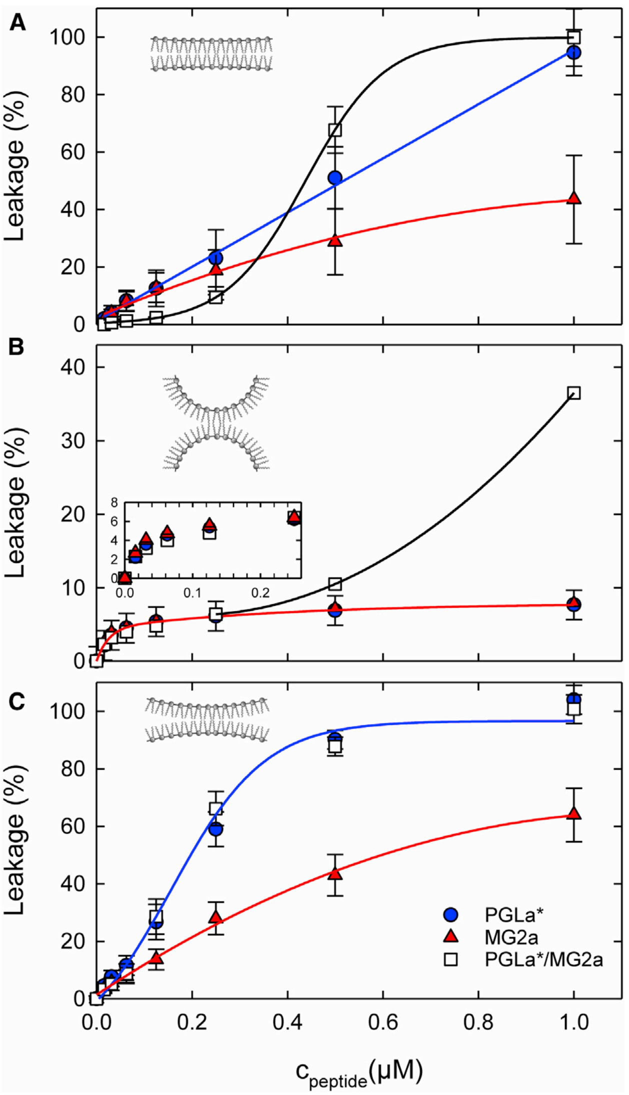

FIGURE 2 Leakage of POPC/POPG (3:1 mol/mol) (*A*), POPE/POPG (3:1 mol/mol) (*B*), and lyso-PE/POPE/POPG (1.6:1.4:1 mol/mol/mol) (*C*) LUVs as a function of L18W-PGLa and MG2a concentration, as well as their equimolar mixture. The lipid concentration was 50 μM. Experimental uncertainties are determined from three independent measurements. The solid lines are guides to the eye. The membrane insets in each panel represent the monolayer curvatures as determined in Table 1. To see this figure in color, go online.

which was reproduced in several replicas. The focus of the present report is at higher concentrations, however. Here, the peptide mixture was more active than L18W-PGLa and MG2a alone for $c_{peptide} \gtrsim 0.4$ μM. At $c_{peptide} =$ 1 μM, leakage induced by just L18W-PGLa was within an experimental resolution equal to that of L18W-PGLa/MG2a.

In POPE/POPG vesicles, in turn, all studied combinations of peptides caused identical leakage ($\leq$6%) up to $c_{peptide} \approx$ 0.25 μM and remained at the same level for the individual peptides at higher concentrations (Fig. 2 *B*). The peptide mixture, however, exhibited a strong increase of efficacy and was about six times more active than L18W-PGLa and MG2a alone at 1 μM. To see whether the different AMP activities are related to intrinsic curvature, we repeated the leakage assay for lyso-PE/POPE/POPG, which has the same lipid headgroup composition as POPE/POPG but $J_0^{mix} \sim 0$, just as POPC/POPG. Indeed, the peptides regained membrane-permeabilizing efficacy, with L18W-PGLa and L18W-PGLa/MG2a causing full LUV leakage at 1 μM concentration, just as observed for POPC/POPG (Fig. 2 *C*).

For mammalian mimics, in turn, our experiments revealed low activity for all peptides. L18W-PGLa exhibited the highest activity, although its maximal leakage did not exceed 10% within the studied concentration range for POPC (Fig. 3 *A*). The activity of the L18W-PGLa/MG2a was between that of L18W-PGLa and MG2a at all concentrations. Most likely this is related to the reduced number of L18W-PGLa molecules in the peptide mixture. Introducing cholesterol, which shifts J_0^{mix} to negative values, did not lead to an increase of activity of L18W-PGLa/MG2a relative to the peptide monomers as in POPE/POPG. Instead, the overall leakage dropped for all peptides almost to the noise level of the measurement (Fig. 3 *B*).

The diverse activities of the presently studied peptides can be summarized by the definition of a synergy parameter. For antimicrobial activity, this is usually based on the MIC (30). In analogy, we define

$$\Sigma = \frac{L_{L18W-PGLa} + L_{MG2a}}{L_{L18W-PGLa/MG2a}}, \tag{1}$$

where $L_{L18W\text{-}PGLa}$ and L_{MG2a} are the leakage values for the individual peptides at half of the peptide concentration used to determine the leakage $L_{L18W\text{-}PGLa/MG2a}$ of their mixture. Hence, $\Sigma < 0.5$ if both peptides act synergistically. Table 1 lists the results for the highest peptide concentration measured. Except for POPE/POPG, all model systems exhibited $\Sigma > 0.5$. This is consistent with a recent report on an increase of PGLa/MG2 synergy for PE/PG mixtures as compared to PC/PG (31). We thus conclude that L18W-PGLa/MG2a act synergistically only in POPE/POPG.

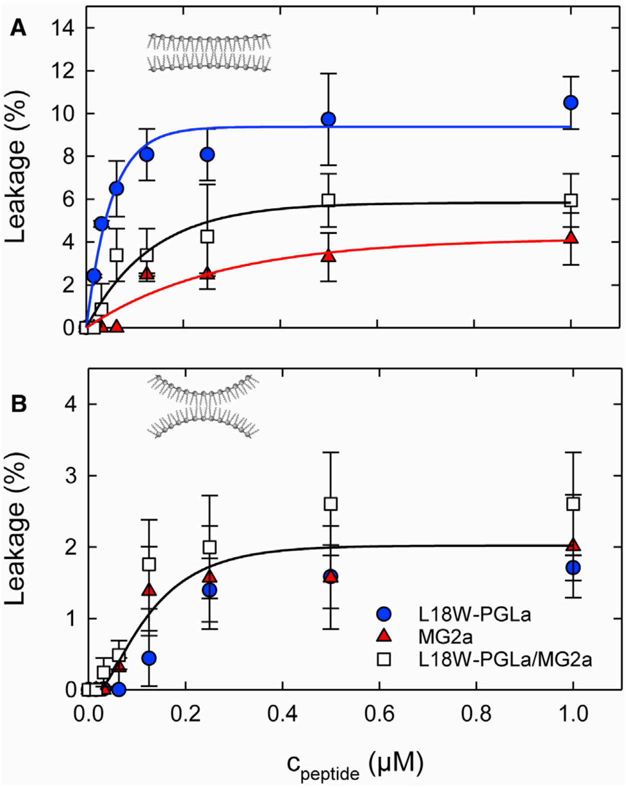

FIGURE 3 Leakage of POPC (*A*) and POPC/Chol (3:1 mol/mol) (*B*) LUVs as a function of L18W-PGLa, MG2a, and their equimolar mixture. The lipid concentration was 50 μM. Experimental uncertainties are determined from three independent measurements. The solid lines are guides to the eye. The membrane insets in each panel represent the monolayer curvatures as determined in Table 1. To see this figure in color, go online.

Effect of peptide dimerization

Synergistic effects of L18W-PGLa and MG2a have been proposed to result from pairwise peptide interaction in membranes (13). Following (16), we therefore constructed L18W-PGLa-MG2a heterodimers using L18W-PGL-GGCa and MG2-GGCa (Fig. 1). Further, L18W-PGL-GGCa and MG2-GGCa form in buffer solution (pH $\sim$7) homodimers (15) as verified by SDS-PAGE (Fig. S2). This enabled us to compare the activities of the diverse magainin dimers. Fig. 4 *A* shows results from leakage experiments on POPE/POPG vesicles. In general, all dimers were able to permeabilize lipid bilayers more strongly than the equimolar mixture of L18W-PGLa and MG2a. More specifically, homodimers of L18W-PGLa and MG2a exhibited similar activities for $c_{peptide} < 0.1$ μM. At higher peptide concentrations, homodimers of L18W-PGLa exhibited a sigmoidal increase reaching nearly 100% leakage, whereas the activity of MG2a homodimers saturated at much lower leakage values ($\sim$60%). Turning to L18W-PGLa-MG2a heterodimers, we observed leakage superior to L18W-PGLa and MG2a homodimers already at

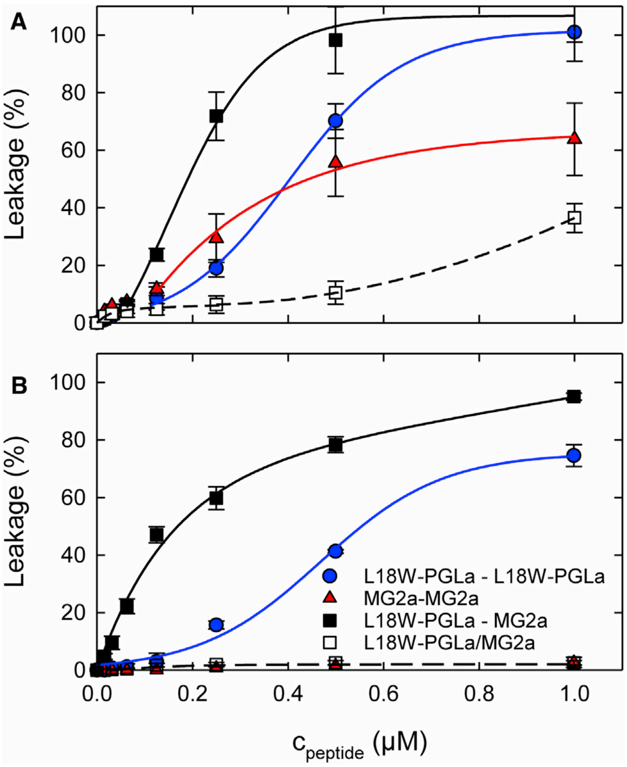

FIGURE 4 Dye release induced by L18W-PGLa/MG2a homodimers and heterodimers. (*A*) compares the activities of L18W-PGLa-L18W-PGLa and MG2a-MG2a homodimers, L18W-PGLa-MG2a heterodimers, and L18W-PGLa/MG2a equimolar mixtures in POPE/POPG LUVs. (*B*) presents results for the same peptides in POPC/Chol LUVs. Note that the results for MG2a homodimers and L18W-PGLa/MG2a are identical within experimental resolution. The lipid concentration was 50 μM. The lines are guides to the eye. To see this figure in color, go online.

low peptide concentration. Final leakage values at $c_{peptide} = 1$ μM were comparable to those of L18W-PGLa homodimers, however.

In the POPC/Chol mixture, effects were significantly different (Fig. 4 *B*). L18W-PGLa-MG2a heterodimers successfully permeabilized the LUVs and induced close to 100% leakage at 1 μM peptide concentration. L18W-PGLa homodimers were active above concentrations of 0.1 μM but reached only ~74% leakage at the highest concentration studied. In contrast, MG2a homodimers, just like L18W-PGLa/MG2a equimolar mixtures, were not able to induce significant dye release from the vesicles. Finally, we performed a control experiment on POPE/POPG vesicles by adding DTT (40 μM), which is known to reduce disulfide bonds (32), to the L18W-PGLa-L18W-PGLa solution before mixing the peptides with LUVs. This in situ transformation of L18W-PGLa homodimers to monomers resulted in permeabilization capabilities identical with that of L18W-PGLa peptide (Fig. S3). Thus, the membrane-lytic activity of L18W-PGLa can be increased strongly by chemical fixation as dimers.

Free energy of peptide translocation

To get deeper insight on the influence of intrinsic curvature on peptide insertion, we performed coarse-grained MC simulations on the translocation of an MG2a mimic through lipid bilayers of different J_0. The sign of intrinsic curvatures of the simulated systems corresponded to those of the measured systems and also included a system of positive intrinsic curvature (see Supporting Material). Simulations clearly show that membrane composition affects the free-energy profiles (Fig. 5). In particular, the system with the most negative J_0 exhibits the highest free-energy penalty for translocation of the peptide across the membrane. The barrier decreases significantly with shifting J_0 toward positive values, which makes the peptide translocation much easier. The translocation is mainly determined by the insertion step, which requires a change in orientation from a parallel to perpendicular/tilted peptide alignment with respect to the membrane surface. Note that the free-energy minimum corresponds to the peptide position in the membrane adsorbed state, which shifts progressively toward the membrane center with increasing J_0, i.e., positive curvature.

Antimicrobial activity of peptide monomers and dimers against *E. coli*

To correlate our results on membrane mimetics to biological activities toward bacteria, we determined the effect of peptides on *E. coli* growth. We observed distinct MIC values for the individual peptides (Fig. 6). In agreement with (16,31), we found synergistic activity of L18W-PGLa/MG2 mixtures ($\Sigma \sim 0.25$) as compared to single-peptide applications. Further, chemically fixed peptide homodimers were significantly more active than their

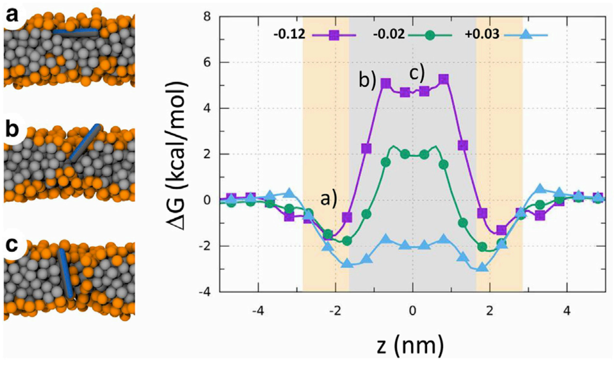

FIGURE 5 Calculated free energy profiles of peptide translocation across membranes with different intrinsic curvature (numbers in legend correspond to J_0 in nm^{-1}). The headgroup and tail regions are shown as orange and gray areas, respectively. The left-hand side shows simulation screen shots of the most important steps of peptide translocation: (*a*) surface adsorption, (*b*) peptide tilting, and (*c*) peptide insertion. To see this figure in color, go online.

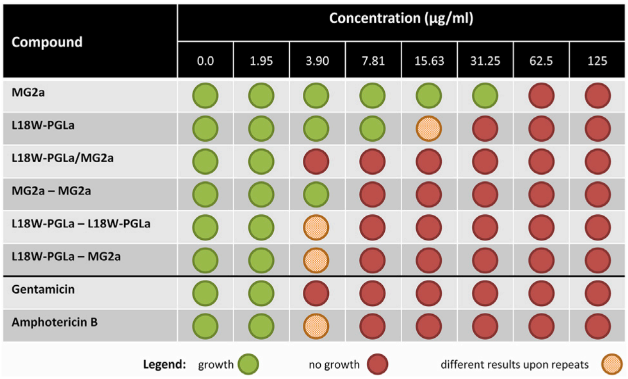

FIGURE 6 Effect of different magainins on the growth of *E. coli* K12. The antibiotics gentamicin and amphotericin B serve as positive controls. To see this figure in color, go online.

monomers. MG2a dimers reduced the MIC from 62.5 to 7.8 μg/mL and L18W-PGLa dimers from ~31.25 to ~7.8 μg/mL, respectively. In particular, L18W-PGLa homodimers exhibited the same efficacy as L18W-PGLa-MG2a heterodimers.

The MIC values can be compared qualitatively to our dye-release experiments on vesicles using the leakage intensities observed at the highest-measured peptide concentrations. Notably, the synergistic activity of the equimolar peptide mixture (Fig. 2 *B*), the similar activities of L18W-PGLa homodimers and L18W-PGLa-MG2a (Fig. 4 *A*), and the high efficacy of L18W-PGLa homodimers compared to individual L18W-PGLa peptide applications (Fig. S3) are well-captured by the POPE/POPG (3:1 mol/mol) mimic. Other features, such as the different antimicrobial activities of L18W-PGLa and MG2, as well as the almost equal efficacy of equimolar peptide mixtures and L18W-PGLa-MG2a hybrids, are not observed in our simple *E. coli* inner membrane mimics, which may be explained by the vastly more complex structure of the *E. coli* cell envelope that consists of an outer membrane, periplasm, and inner membrane. We emphasize, however, that the synergistic activity of L18W-PGLa and MG2a, which is the main focus of this work, is reproduced in POPE/POPG 3:1 (mol/mol).

We can further compare our leakage results on POPC/Chol vesicles to the peptide's hemolytic activity. Nishida et al. (16) reported a sixfold increase of erythrocyte hemolysis for PGLa-MG2a heterodimers as compared to the PGLa/MG2a equimolar mixture. Further, compared to single peptides, the heterodimer's hemolytic activity was even 40- to 60-fold increased. This latter result correlates qualitatively with our findings in POPC/Chol (3:1 mol/mol) vesicles, in which the heterodimers showed a 50 times higher activity than the L18W-PGLa/MG2a mixture (Fig. 4 *B*). Hence, POPC/Chol appears to be a reasonable first-order lipid-only model to estimate the hemolytic potential of magainins.

DISCUSSION

Synergism is coupled to negative intrinsic curvature

Coupling experimental data for intrinsic curvatures and AMP-induced ANTS/DPX dye release from selected lipid-only mimics of bacterial and mammalian membranes provided clear evidence for the delicate balance of membrane curvature elasticity, overall bilayer surface charge, and peptide properties in promoting or obstructing the synergistic activity of L18W-PGLa/MG2a mixtures. The synergism of PGLa and MG2 has been studied before by leakage assays on diverse lipid vesicles, showing significant enhancement of peptide activity for PGLa/MG2 equimolar mixtures (13,33).

Pronounced synergism was observed only in POPE/POPG bilayers (Fig. 2 *B*). The requirement of high peptide concentration to permeabilize PE-enriched bilayers may be additionally enhanced by extensive intermolecular H-bonding of PEs (34,35). Most interestingly, however, L18W-PGLa/MG2a synergism is fully abolished in lyso-PE/POPE/POPG (Fig. 2 *C*). This system was designed to have 1) the same overall charge as POPC/POPG and POPE/POPG, 2) the same headgroup composition as POPE/POPG (i.e., the same abilities to form H-bonds), and 3) the overall same intrinsic curvature as POPC/POPG (J_0~0). The loss of synergism in lyso-PE/POPE/POPG hence provides unambiguous evidence for a coupling to negative intrinsic curvature.

It has been recognized for some time that protein/peptide activity couples to intrinsic lipid curvatures (36–38). Intrinsic lipid curvature is a property of lipid monolayers (i.e., each membrane leaflet) that gives rise to an elastic energy stored in flat lipid bilayers (see, e.g., (39)), which may be released upon interaction with a membrane active compound (36). That is, lateral strain, in particular at the polar/apolar interface, is significantly larger for lipids with a more negative J_0, whereas lipid membranes with a positive J_0 have a looser polar/apolar interface. Membrane intrinsic curvature has been previously related to MG2 activity (40) or synergism (19). Conversely, the latter authors concluded that synergistic interactions between PGLa/MG2 are related to positive J_0 that would allow the peptides to penetrate into the bilayer more easily (19). This assessment was based on the assumption that synergism is related to the adoption of a tilted or inserted PGLa topology in the presence of MG2. This hypothesis has been cast into doubt by (41,42), which show that the peptides develop synergistic activities in an in-planar topology. Strandberg et al. (19) also notably reported mainly surface aligned PGLa/MG2 for POPC/POPG bilayers, whereas MG2-mediated PGLa insertion was reported only for lipid mixtures with disaturated hydrocarbon chains (17,19).

Based on our results, we agree with (17,19) that membranes with a more positive curvature facilitate peptide insertion into the bilayer interface because of reduced lateral stress at the bilayer's polar/apolar interface (i.e., looser

packing). However, at the same time, this hampers the development of synergy because both magainins are able to disrupt the polar/apolar interface at low energetic cost. POPE/POPG (3:1 mol/mol), in turn, displays substantial interfacial stress, and indeed both magainins did not induce significant vesicle leakage (Fig. 2 *B*). However, when applied as an equimolar mixture, they clearly showed synergy with $\Sigma = 0.4$ (Table 1), which is in good agreement with recent observations from Zerweck et al. (31). Corroborated by our MC simulations (Fig. 5), these findings strongly suggest an activation barrier for vesicle leakage, $\Delta G^{\ddagger}$, that is mediated by the interfacial packing density and hence lipid composition. In addition, synergy requires anionic lipids for increased affinity of peptides to the membrane surface. This is clearly demonstrated by POPC/Chol, which contains significant intrinsic negative curvature energies but resulted in a synergy parameter $\Sigma = 1.2$ (Table 1).

To appreciate the different activities of L18W-PGLa and MG2a, it is instructive to consider the energetics of membrane insertion based on the Wimley-White hydrophobicity scale (43). We thus calculated estimates for the Gibbs free energy of insertion into the bilayer nonpolar region (44)

$$\Delta G^o_{ins} \approx \Delta G^o_f + \Delta G^o_{oct} - \Delta G^o_{if} \approx \Delta G^o_{oct} - \Delta G^o_{if} = \Delta G^o_{oct-if}, \quad (2)$$

using the Membrane Protein Explorer (MPEx, http://blanco.biomol.uci.edu/mpex/), where ΔG^o_f is the free energy from peptide conformational changes in the aqueous phase (assumed to be insignificant), ΔG^o_{oct} is the free energy of peptide transfer from water to octanol, and ΔG^o_{if} is the free energy of binding to the membrane-water interface. Calculation of ΔG^o_{if} requires knowledge of the peptide's helicity taken up at the membrane surface. For charged membranes, electrostatic interactions need to be considered (45). However, corrections calculated via the Gouy-Chapman theory for melittin yielded the same surface-partition coefficient for charged and charge-neutral bilayers (45). For our semiquantitative discussion, it thus suffices to derive ΔG^o_{if} based on the peptide's helicity in PC bilayers (Table 2). Our ΔG^o_{ins} estimates show that L18W-PGLa has a higher propensity for being located within the membrane core than MG2a. This agrees with previous NMR data showing that PGLa may tilt into the bilayer core, whereas MG2 always remained parallel at the membrane surface (18,19).

TABLE 2 Thermodynamics of AMP Insertion in PC Membranes According to the Wimley-White Hydrophobicity Scale

	% helix in PC	ΔG^o_{if} (kcal/mol)	ΔG^o_{oct} (kcal/mol)	ΔG^o_{ins} (kcal/mol)	μ_H
MG2a	83[a]	−7.3	16.9	24.2	14.1
L18W-PGLa	72[b]	−6.1	16.2	22.3	10.9

The Wimley-White hydrophobicity scale is from Ref. (43).
[a]Taken from (66).
[b]Taken from (67).

To correlate these considerations with our leakage data, it is further necessary to consider $\Delta G^{\ddagger}$ as introduced above. Inspired by (44), we can draw a hypothetical energy path for AMP insertion and synergism (Fig. 7). In particular, the difference in ΔG_{if} between L18W-PGLa and MG2a (Table 2) should lead to a higher $\Delta G^{\ddagger}$ for MG2a in bilayers of equal lipid composition, which would explain why MG2a is always less active than L18W-PGLa. In the case of synergistic interaction between L18W-PGLa and MG2a, the activation barrier also appears to be lowered. We speculate in particular that the perturbation of the lipid bilayer by L18W-PGLa helps to lower $\Delta G^{\ddagger}$ for MG2a, which, because of its higher mean hydrophobic moment μ_H (Table 2), i.e., amphipathicity, will cause a stronger impairment of the bilayer's permeability barrier because of a larger mismatch with the polarity profile of the lipid bilayer. This agrees with a previous analysis of properties of α-helical antimicrobial peptides showing that their efficacy is dominated by their overall amphipathicity (46). Note that previous solid-state NMR measurements did not report on PGLa-mediated insertion of MG2 (18,19). However, detection of such states could have been impeded by a limited degree of sample hydration, which has been shown previously to significantly increase threshold concentrations for peptide insertion into lipid bilayers (see, e.g., (47)).

Synergism is not necessarily coupled to L18W-PGLa/MG2a pair formation

Motivated by a maximum of antimicrobial activity at equimolar peptide ratio, a number of studies claim that

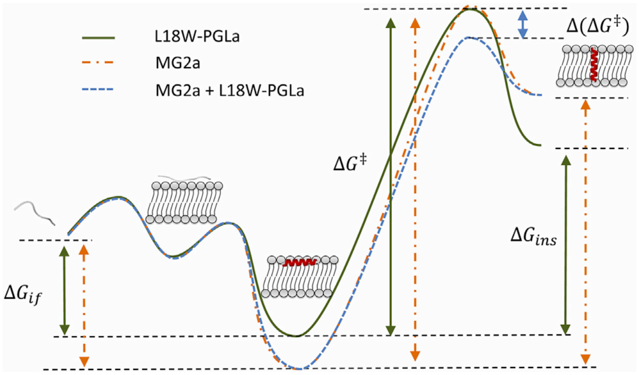

FIGURE 7 Schematic free-energy landscape with different states during the peptide-insertion process into a lipid bilayer, including adsorption to and folding at the membrane surface ΔG_{if}, as well as insertion into the hydrophobic core ΔG_{ins}, for which the peptide has to overcome an activation barrier $\Delta G^{\ddagger}$. Depending on the energy difference between the transition state (‡) and the peptide's interfacial state, $\Delta G^{\ddagger}$ can vary significantly. ΔG_{if} is assumed to be smaller for L18W-PGLa than for MG2a. Synergistic interactions between the peptides may lower the barrier by $\Delta(\Delta G^{\ddagger})$. To see this figure in color, go online.

synergism of magainins is due to a pairwise interaction of the two AMPs (formation of physical heterodimers) in the presence of the lipid bilayer (13,15,16,33). Further, molecular dynamics simulations (48) and mutation studies (31) suggest that close contact interactions between glycine and alanine residues on PGLa support the formation of an antiparallel (physical) homodimer. Indeed, chemically cross-linked L18W-PGLa homodimers are almost as active as L18W-PGLa-MG2a heterodimers in POPE/POPG (Fig. 4 *A*). These results are in good agreement with several previous studies demonstrating that peptide aggregates (dimers) are in general—due to their larger size—significantly more perturbing bilayers than their monomers (see, e.g., (49–52)). Nishida et al. (16) argued that association of L18W-PGLa and MG2a is concentration-dependent and that cysteine-linked L18W-PGLa-MG2a mimic high peptide concentrations. However, even at our highest peptide concentration, which is ∼5 times larger than that applied in (16), the heterodimers are still 2–3 times more effective than the equimolar L18W-PGLa/MG2a mixture. Considering that peptides diffuse in membranes at a similar rate to lipids (53,54), it thus occurs that L18W-PGLa/MG2a pairs should have formed rapidly on the time scale of our experiment. Thus, aggregation of L18W-PGLa and MG2a is not a strict prerequisite for developing synergism. In turn, our conclusion is in agreement with (41), who found that carboxyterminal-GGS or -GGA analogs of PGLa and MG2 are more active than PGLa/MG2a mixtures, although they are not able to form cysteine cross-links.

Correlation of membrane-lytic activities of magainins between plasma membrane mimics and live cells

The role of membrane lipid architecture in AMP selectivity has been extensively discussed before (see, e.g., (29)). One of the most obvious differences in lipid architecture is the absence of charged lipid species in the outer leaflet of mammalian plasma membranes, whereas bacterial plasma membranes contain anionic lipid species. Thus, attractive electrostatic interactions between cationic residues on AMP and anionic lipids are an essential first step of discrimination that has been already recognized for several years (for review, see, e.g., (6)). However, the effects of membrane elasticity in particular at the polar/apolar interface are of similar importance to consider. This is convincingly demonstrated by comparing the synergistic peptide activities found for POPE/POPG with the peptide activity on *E. coli*. For POPC/POPG, in turn, we found no such agreement. Moreover, the increased activity of L18W-PGLa-MG2a in POPC/Chol mixtures correlates well with their increased hemolysis of erythrocytes (16). We note, however, that other aspects, such as differences in L18W-PGLa and MG2a activities or the almost-equal killing efficacy of equimolar L18W-PGLa/MG2a mixtures and L18W-PGLa-MG2a heterodimers, are not mirrored in our leakage experiments on POPE/POPG (3:1 mol/mol) LUVs. This indicates limitations of the currently applied lipid-only model systems, which are unable to capture the full complexity of the *E. coli* cell envelope. We are currently developing more complex membrane mimetic systems.

CONCLUSION

We have provided experimental evidence for the significance of combining a negatively charged membrane surface with a tightly packed polar/apolar interface for synergistic interactions of L18W-PGLa and MG2a with lipid membranes. Tight packing of this interface is achieved here by POPE, which has a significant negative intrinsic curvature. Cardiolipin, which is also present in cytoplasmic membranes of Gram-negative bacteria (20) and which has negative J_0 in the presence of divalent ions (22), may further add to the interfacial packing strain. If bilayers have negligible overall intrinsic curvature, L18W-PGLa and MG2a permeabilize the membrane much more easily and do not develop synergism. This effect is expected to be even more pronounced for lipids with positive intrinsic curvature, which agrees well with previous observations (18,19). Consequently, “softening up” the polar/apolar interface by a concerted action of MG2a and L18W-PGLa appears to be the underlying mechanism of synergism for the two peptides. We speculate that L18W-PGLa is the “helper molecule” that preconditions the bilayer and thereby allows MG2a, which, due to its more pronounced amphipathicity, is less compatible with the distribution of polar and nonpolar moieties in lipid membranes, to increase its activity. In addition, synergism also requires electrostatic interactions between the peptides and membrane lipids to anchor the AMPs more strongly within the membrane interface. Importantly, however, pairwise interactions of L18W-PGLa and MG2a are not strictly required for this scenario, which is supported by our experiments on homo- and heterodimers of the two peptides and which agrees with a recent report (41). Additional effects, such as peptide-induced lipid phase separation, may further influence this mechanism. Corresponding experiments will be performed in our laboratory in the near future. The correlation of synergistic activity of L18W-PGLa and MG2a in *E. coli* with dye-release experiments in POPE/POPG LUVs but the inability to reproduce other features of AMP activity in bacteria strongly supports a proper choice of the lipid mimetic system for unraveling the biophysics of AMP-membrane interactions.

SUPPORTING MATERIAL

Supporting Materials and Methods, five figures, and two tables are available at http://www.biophysj.org/biophysj/supplemental/S0006-3495(18)30323-0.

AUTHOR CONTRIBUTIONS

R.L. performed research, analyzed data, and wrote the article. M.P. performed research and analyzed data. I.K. performed MC simulations. I.S. performed experiments on *E. coli*. D.E. performed research. R.V., G.P., and K.L. designed research and wrote the article.

ACKNOWLEDGMENTS

The authors thank Christopher Aisenbrey and Burkhard Bechinger for stimulating discussions.

This work was supported by the Austrian Science Fund FWF, project No. I 1763-B21 (to K.L.), as well as the Czech Science Foundation (grant 17-11571S to R.V.) and the Central European Institute of Technology 2020 (LQ1601) project, with financial contribution made by the Ministry of Education, Youths and Sports of the Czech Republic and special support paid from the National Programme for Sustainability II funds. Computational resources were provided by the CESNET LM2015042 and the IT4Innovations National Supercomputing Center–LM2015070, provided under the program Projects of Large Research, Development, and Innovations Infrastructures.

SUPPORTING CITATIONS

References (55–65) appear in the Supporting Material.

REFERENCES

1. Mayer, M. L., D. M. Easton, and R. E. W. Hancock. 2010. Fine tuning host responses in the face of infection: emerging roles and clinical applications of host defence peptides. *In* Antimicrobial Peptides: Discovery, Design and Novel Therapeutic Strategies. G. Wang, ed. CABI, pp. 195–220.
2. Steinstraesser, L., U. Kraneburg, …, S. Al-Benna. 2011. Host defense peptides and their antimicrobial-immunomodulatory duality. *Immunobiology.* 216:322–333.
3. Hancock, R. E., E. F. Haney, and E. E. Gill. 2016. The immunology of host defence peptides: beyond antimicrobial activity. *Nat. Rev. Immunol.* 16:321–334.
4. Wimley, W. C., and K. Hristova. 2011. Antimicrobial peptides: successes, challenges and unanswered questions. *J. Membr. Biol.* 239:27–34.
5. Lohner, K. 2017. Membrane-active antimicrobial peptides as template structures for novel antibiotic agents. *Curr. Top. Med. Chem.* 17:508–519.
6. Lohner, K., and S. E. Blondelle. 2005. Molecular mechanisms of membrane perturbation by antimicrobial peptides and the use of biophysical studies in the design of novel peptide antibiotics. *Comb. Chem. High Throughput Screen.* 8:241–256.
7. Henderson, J. M., and K. Y. C. Lee. 2013. Promising antimicrobial agents designed from natural peptide templates. *Curr. Opin. Solid State Mater. Sci.* 17:175–192.
8. Hoffmann, W., K. Richter, and G. Kreil. 1983. A novel peptide designated PYLa and its precursor as predicted from cloned mRNA of Xenopus laevis skin. *EMBO J.* 2:711–714.
9. Soravia, E., G. Martini, and M. Zasloff. 1988. Antimicrobial properties of peptides from Xenopus granular gland secretions. *FEBS Lett.* 228:337–340.
10. Zasloff, M. 1987. Magainins, a class of antimicrobial peptides from Xenopus skin: isolation, characterization of two active forms, and partial cDNA sequence of a precursor. *Proc. Natl. Acad. Sci. USA.* 84:5449–5453.
11. Williams, R. W., R. Starman, …, D. Covell. 1990. Raman spectroscopy of synthetic antimicrobial frog peptides magainin 2a and PGLa. *Biochemistry.* 29:4490–4496.
12. Westerhoff, H. V., M. Zasloff, …, D. Juretić. 1995. Functional synergism of the magainins PGLa and magainin-2 in Escherichia coli, tumor cells and liposomes. *Eur. J. Biochem.* 228:257–264.
13. Matsuzaki, K., Y. Mitani, …, K. Miyajima. 1998. Mechanism of synergism between antimicrobial peptides magainin 2 and PGLa. *Biochemistry.* 37:15144–15153.
14. Wieprecht, T., O. Apostolov, …, J. Seelig. 2000. Membrane binding and pore formation of the antibacterial peptide PGLa: thermodynamic and mechanistic aspects. *Biochemistry.* 39:442–452.
15. Hara, T., Y. Mitani, …, K. Matsuzaki. 2001. Heterodimer formation between the antimicrobial peptides magainin 2 and PGLa in lipid bilayers: a cross-linking study. *Biochemistry.* 40:12395–12399.
16. Nishida, M., Y. Imura, …, K. Matsuzaki. 2007. Interaction of a magainin-PGLa hybrid peptide with membranes: insight into the mechanism of synergism. *Biochemistry.* 46:14284–14290.
17. Tremouilhac, P., E. Strandberg, …, A. S. Ulrich. 2006. Synergistic transmembrane alignment of the antimicrobial heterodimer PGLa/magainin. *J. Biol. Chem.* 281:32089–32094.
18. Salnikov, E. S., and B. Bechinger. 2011. Lipid-controlled peptide topology and interactions in bilayers: structural insights into the synergistic enhancement of the antimicrobial activities of PGLa and magainin 2. *Biophys. J.* 100:1473–1480.
19. Strandberg, E., J. Zerweck, …, A. S. Ulrich. 2013. Synergistic insertion of antimicrobial magainin-family peptides in membranes depends on the lipid spontaneous curvature. *Biophys. J.* 104:L9–L11.
20. Wilkinson, S. G. 1988. Gram-negative bacteria. *In* Microbial Lipids S. G. Wilkinson, and C. Ratledge, eds. . Academic Press, pp. 299–488.
21. Morein, S., A. Andersson, …, G. Lindblom. 1996. Wild-type Escherichia coli cells regulate the membrane lipid composition in a “window” between gel and non-lamellar structures. *J. Biol. Chem.* 271:6801–6809.
22. Chen, Y. F., K. Y. Tsang, …, Z. A. Fan. 2015. Differential dependencies on [Ca2+] and temperature of the monolayer spontaneous curvatures of DOPE, DOPA and cardiolipin: effects of modulating the strength of the inter-headgroup repulsion. *Soft Matter.* 11:4041–4053.
23. Zweytick, D., G. Deutsch, …, K. Lohner. 2011. Studies on lactoferricin-derived Escherichia coli membrane-active peptides reveal differences in the mechanism of N-acylated versus nonacylated peptides. *J. Biol. Chem.* 286:21266–21276.
24. Kollmitzer, B., P. Heftberger, …, G. Pabst. 2013. Monolayer spontaneous curvature of raft-forming membrane lipids. *Soft Matter.* 9:10877–10884.
25. Cooke, I. R., K. Kremer, and M. Deserno. 2005. Tunable generic model for fluid bilayer membranes. *Phys. Rev. E Stat. Nonlin. Soft Matter Phys.* 72:011506.
26. Vácha, R., and D. Frenkel. 2011. Relation between molecular shape and the morphology of self-assembling aggregates: a simulation study. *Biophys. J.* 101:1432–1439.
27. Wang, F., and D. P. Landau. 2001. Efficient, multiple-range random walk algorithm to calculate the density of states. *Phys. Rev. Lett.* 86:2050–2053.
28. Marsh, D. 1996. Intrinsic curvature in normal and inverted lipid structures and in membranes. *Biophys. J.* 70:2248–2255.
29. Lohner, K., E. Sevcsik, and G. Pabst. 2008. Liposome-based biomembrane mimetic systems: implications for lipid-peptide interactions. *In* Advances in Planar Lipid Bilayers and Liposomes (Vol. 6). A. Leitmannova-Liu, ed. Elsevier, pp. 103–137.
30. Meletiadis, J., S. Pournaras, …, T. J. Walsh. 2010. Defining fractional inhibitory concentration index cutoffs for additive interactions based on self-drug additive combinations, Monte Carlo simulation analysis, and in vitro-in vivo correlation data for antifungal drug combinations against Aspergillus fumigatus. *Antimicrob. Agents Chemother.* 54:602–609.

31. Zerweck, J., E. Strandberg, …, A. S. Ulrich. 2017. Molecular mechanism of synergy between the antimicrobial peptides PGLa and magainin 2. *Sci. Rep.* 7:13153.

32. Liang, H. P., T. M. Brophy, and P. J. Hogg. 2011. Redox properties of the tissue factor Cys186-Cys209 disulfide bond. *Biochem. J.* 437:455–460.

33. Zerweck, J., E. Strandberg, …, A. S. Ulrich. 2016. Homo- and heteromeric interaction strengths of the synergistic antimicrobial peptides PGLa and magainin 2 in membranes. *Eur. Biophys. J.* 45:535–547.

34. Boggs, J. M. 1980. Intermolecular hydrogen bonding between lipids: influence on organization and function of lipids in membranes. *Can. J. Biochem.* 58:755–770.

35. Pink, D. A., S. McNeil, …, M. J. Zuckermann. 1998. A model of hydrogen bond formation in phosphatidylethanolamine bilayers. *Biochim. Biophys. Acta.* 1368:289–305.

36. Koller, D., and K. Lohner. 2014. The role of spontaneous lipid curvature in the interaction of interfacially active peptides with membranes. *Biochim. Biophys. Acta.* 1838:2250–2259.

37. Dan, N., and S. A. Safran. 1998. Effect of lipid characteristics on the structure of transmembrane proteins. *Biophys. J.* 75:1410–1414.

38. Botelho, A. V., N. J. Gibson, …, M. F. Brown. 2002. Conformational energetics of rhodopsin modulated by nonlamellar-forming lipids. *Biochemistry.* 41:6354–6368.

39. Shearman, G. C., O. Ces, …, J. M. Seddon. 2006. Inverse lyotropic phases of lipids and membrane curvature. *J. Phys. Condens. Matter.* 18:S1105–S1124.

40. Matsuzaki, K., K. Sugishita, …, R. M. Epand. 1998. Relationship of membrane curvature to the formation of pores by magainin 2. *Biochemistry.* 37:11856–11863.

41. Glattard, E., E. S. Salnikov, …, B. Bechinger. 2016. Investigations of the synergistic enhancement of antimicrobial activity in mixtures of magainin 2 and PGLa. *Biophys. Chem.* 210:35–44.

42. Marquette, A., E. S. Salnikov, …, B. Bechinger. 2016. Magainin 2-PGLa interactions in membranes - two peptides that exhibit synergistic enhancement of antimicrobial activity. *Curr. Top. Med. Chem.* 16:65–75.

43. White, S. H., and W. C. Wimley. 1999. Membrane protein folding and stability: physical principles. *Annu. Rev. Biophys. Biomol. Struct.* 28:319–365.

44. Almeida, P. F., and A. Pokorny. 2009. Mechanisms of antimicrobial, cytolytic, and cell-penetrating peptides: from kinetics to thermodynamics. *Biochemistry.* 48:8083–8093.

45. Beschiaschvili, G., and J. Seelig. 1990. Melittin binding to mixed phosphatidylglycerol/phosphatidylcholine membranes. *Biochemistry.* 29:52–58.

46. Pathak, N., R. Salas-Auvert, …, R. G. Harrison. 1995. Comparison of the effects of hydrophobicity, amphiphilicity, and alpha-helicity on the activities of antimicrobial peptides. *Proteins.* 22:182–186.

47. Huang, H. W., and Y. Wu. 1991. Lipid-alamethicin interactions influence alamethicin orientation. *Biophys. J.* 60:1079–1087.

48. Ulmschneider, J. P., J. C. Smith, …, E. Strandberg. 2012. Reorientation and dimerization of the membrane-bound antimicrobial peptide PGLa from microsecond all-atom MD simulations. *Biophys. J.* 103:472–482.

49. Takei, J., A. Remenyi, and C. E. Dempsey. 1999. Generalised bilayer perturbation from peptide helix dimerisation at membrane surfaces: vesicle lysis induced by disulphide-dimerised melittin analogues. *FEBS Lett.* 442:11–14.

50. Dempsey, C. E., S. Ueno, and M. B. Avison. 2003. Enhanced membrane permeabilization and antibacterial activity of a disulfide-dimerized magainin analogue. *Biochemistry.* 42:402–409.

51. Lorenzón, E. N., N. A. Santos-Filho, …, E. M. Cilli. 2016. C-terminal lysine-linked magainin 2 with increased activity against multidrug-resistant bacteria. *Protein Pept. Lett.* 23:738–747.

52. Verly, R. M., J. M. Resende, …, B. Bechinger. 2017. Structure and membrane interactions of the homodimeric antibiotic peptide homotarsinin. *Sci. Rep.* 7:40854.

53. Frey, S., and L. K. Tamm. 1990. Membrane insertion and lateral diffusion of fluorescence-labelled cytochrome c oxidase subunit IV signal peptide in charged and uncharged phospholipid bilayers. *Biochem. J.* 272:713–719.

54. Gambin, Y., R. Lopez-Esparza, …, W. Urbach. 2006. Lateral mobility of proteins in liquid membranes revisited. *Proc. Natl. Acad. Sci. USA.* 103:2098–2102.

55. Broekhuyse, R. M. 1968. Phospholipids in tissues of the eye. I. Isolation, characterization and quantitative analysis by two-dimensional thin-layer chromatography of diacyl and vinyl-ether phospholipids. *Biochim. Biophys. Acta.* 152:307–315.

56. Buboltz, J. T., and G. W. Feigenson. 1999. A novel strategy for the preparation of liposomes: rapid solvent exchange. *Biochim. Biophys. Acta.* 1417:232–245.

57. Rieder, A. A., D. Koller, …, G. Pabst. 2015. Optimizing rapid solvent exchange preparation of multilamellar vesicles. *Chem. Phys. Lipids.* 186:39–44.

58. Alley, S. H., O. Ces, …, R. H. Templer. 2008. X-ray diffraction measurement of the monolayer spontaneous curvature of dioleoylphosphatidylglycerol. *Chem. Phys. Lipids.* 154:64–67.

59. Leikin, S., M. M. Kozlov, …, R. P. Rand. 1996. Measured effects of diacylglycerol on structural and elastic properties of phospholipid membranes. *Biophys. J.* 71:2623–2632.

60. Israelachvili, J. N. 2011. Intermolecular and Surface Forces, Third Edition. Academic Press, Burlington, MA.

61. Ellens, H., J. Bentz, and F. C. Szoka. 1985. H+- and Ca2+-induced fusion and destabilization of liposomes. *Biochemistry.* 24:3099–3106.

62. Ladokhin, A. S., W. C. Wimley, and S. H. White. 1995. Leakage of membrane vesicle contents: determination of mechanism using fluorescence requenching. *Biophys. J.* 69:1964–1971.

63. Laemmli, U. K. 1970. Cleavage of structural proteins during the assembly of the head of bacteriophage T4. *Nature.* 227:680–685.

64. Frenkel, D., and B. Smit. 2002. Understanding Molecular Simulation. Academic Press, San Diego, CA.

65. Mueller, J. H., and J. Hinton. 1941. A protein-free medium for primary isolation of the gonococcus and meningococcus. *Proc. Soc. Exp. Biol. Med.* 48:330–333.

66. Gesell, J., M. Zasloff, and S. J. Opella. 1997. Two-dimensional 1H NMR experiments show that the 23-residue magainin antibiotic peptide is an α-helix in dodecylphosphocholine micelles, sodium dodecylsulfate micelles, and trifluoroethanol/water solution. *J. Biomol. NMR.* 9:127–135.

67. Bechinger, B., M. Zasloff, and S. J. Opella. 1998. Structure and dynamics of the antibiotic peptide PGLa in membranes by solution and solid-state nuclear magnetic resonance spectroscopy. *Biophys. J.* 74:981–987.

Biophysical Journal, Volume *114*

Supplemental Information

Synergism of Antimicrobial Frog Peptides Couples to Membrane Intrinsic Curvature Strain

Regina Leber, Michael Pachler, Ivo Kabelka, Irene Svoboda, Daniel Enkoller, Robert Vácha, Karl Lohner, and Georg Pabst

Supplementary Information

Synergism of Antimicrobial Frog Peptides Couples to Membrane Intrinsic Curvature Strain

Regina Leber, Michael Pachler, Ivo Kabelka, Irene Svoboda, Daniel Enkoller, Robert Vácha, Karl Lohner, and Georg Pabst

PREPARATION OF LIPOSOMES

Lipid films (20 mg total lipid) for dye-release experiments were prepared by mixing the appropriate amounts of dissolved phospholipids before evaporation of solvents under a stream of nitrogen and stored in vacuum overnight. After addition of Hepes buffer containing the fluorophor/quencher ANTS/DPX (10 mM Hepes, 68 mM NaCl, 12.5 mM ANTS, 45 mM DPX, pH 7.4), formation of lipid vesicles was achieved by equilibration at 15°C above the lipid's melting temperature and intermittent vigorous vortex mixing. PE/PG-mixtures were additionally homogenized by six freeze/thaw-cycles. Large unilamellar vesicles (LUVs) were obtained by 25 cycles of extrusion of the hydrated liposomes through a polycarbonate filter (Millipore-Isopore^{TM}, Merck, Vienna) of 0.1 µm pore size above the lipid's melting temperature. Non-encapsulated fluorophore and quencher were removed by size-exclusion chromatography using SephadexTM G-75 (Sigma-Aldrich, Vienna) gel as described earlier (1). LUVs were eluted with Hepes buffer (10 mM Hepes, 140 mM NaCl, pH 7.4). Liposomal lipids were quantified by the determination of lipid phosphorus according the method described by (2). The final LUV concentration for dye-release experiments was adjusted to 50 µM in Hepes buffer (10 mM Hepes, 140 mM NaCl, pH 7.4).

PREPARATION AND ANALYSIS OF INVERTED HEXAGONAL PHASES BY SAXS

Samples for X-ray analysis were prepared by rapid solvent exchange (RSE) (3) using an adapted method that allows control of temperature (4). Here we focus in particular on the intrinsic curvatures of POPG and lyso-PE, whose values have to the best of our knowledge not been reported before. Both lipids do not form H_{II} phases and were therefore mixed with DOPE at various amounts as detailed above. In brief, appropriate amounts of lipid stock solution were pipetted into test tubes containing NaPi buffer (20 mM Na-phosphate, 130 mM NaCl, pH 7.4) and which were pre-equilibrated at a given temperature. The test tubes were quickly mounted onto the RSE apparatus, setting the argon flow to 60 ml/min and the vortex speed to 1000-1200 rpm. Rapid evaporation was initiated by opening the valve to a vacuum

pump set to a pressure in between the vapor pressure of solvent and water leading to the formation of lipid aggregates. Sample preparation was terminated after six minutes. Details of RSE settings are listed in supplementary Table S1. Control experiments using ^{1}H NMR showed no contamination of the samples with organic solvent.

Table S1: Settings used for the rapid solvent exchange.

Lipids	Temperature [°C]	Pressure [mbar]	Vortex speed [U/min]
DOPE	45	200	1200
POPG/DOPE	60	300	1000
lyso-PE/DOPE	70	900	1000
POPE	55	360	1000

All samples contained 12 wt% tricosene, which is needed to reduce the packing frustration of the inverted hexagonal phase (H_{II}) (see, e.g., (5)). This effect decreases the lamellar-to-H_{II} phase transition of POPE close to room temperature (6). POPG and lyso-PE do not form H_{II} phases. Therefore they were mixed with DOPE at various concentrations using a previously reported protocol (6). Samples containing DOPE and POPG were measured immediately after preparation, while lyso-PE containing samples were equilibrated for three days at room temperature in argon atmosphere to avoid lipid oxidation.

SAXS measurements were performed using a SAXSpace compact camera from Anton Paar (Austria), equipped with a Pilatus 100K-S detector system (Dectris, Switzerland) and a Genix 3D microfocus X-ray generator from Xenocs (Grenoble, France) [beam-size (Cu-Kα): ~ 300 µm (diameter)]. A scattering vector range of q = 0.6 nm^{-1} o 10.9 nm^{-1} was covered by setting the sample-to-detector distance to 308 mm. Temperature control was provided by a Peltier unit to within ± 0.1 °C. All samples were transferred into a paste cell holder (Anton Paar, Austria) and equilibrated for 10 minutes at 35°C before exposing to X-rays for one hour. Data reduction was performed with SAXStreat (Anton Paar, Austria). Note that temperature dependencies of intrinsic curvatures are in the range of $\frac{\Delta J_0}{T} = -1 \times 10^{-3}$ nm^{-1}/K^{-1} to -3×10^{-3} nm^{-1}/K^{-1} (6). Temperature corrections to adjust the here reported J_0's to 37°C used for vesicle leakage assays are consequently within experimental error.

SAXS data analysis was performed according to Kollmitzer et al. (6). In brief, intensities and peak positions of H_{II} phase patterns $I_{h,k}$ were used to construct electron density maps

$$\rho(\vec{r}) = \sum_{h,k} \alpha_{h,k} \sqrt{\frac{I_{h,k} q_{h,k}^2}{m_{h,k}}} \cos\left(2\pi \overrightarrow{q_{h,k}}\, \vec{r}\right), \quad \text{(S1)}$$

where h, k are the Miller indices, $m_{h,k}$ is the multiplicity of equivalent diffraction planes and $\alpha_{h,k}$ is the phase. Subsequently, electron density maps were radially integrated over azimuthal angles between 0° and 60° to yield one-dimensional electron density profiles. The maximum of these profiles was fitted with a Gaussian to determine the position of the lipid headgroup R_p. The intrinsic curvature $J_0 = -1/R_0$ is then determined by finding the position of the neutral plane $R_0 = R_p + d_{H1}$ (7), which is assumed to be located at the glycerol backbone (6). Specifically, we used $d_{H1} = 0.44$ nm as estimate for the distance between the lipid heads and the backbone (6).

The intrinsic curvature of non H_{II}-phase forming lipids was estimated assuming linear additivity using

$$J_0 = \frac{J_0^{mix} - (1-\chi) J_0^{DOPE}}{\chi}, \quad \text{(S2)}$$

where χ is the mole fraction of the respective 'guest' lipid in DOPE, which is used as a H_{II}-phase forming template ('host'). To increase the reliability of the J_0 estimates for guest lipids several guest/host mixtures were measured and interpolated, ensuring full miscibility of the guest lipids within the host structure (Fig. S1) and (6).

Figure S1 shows the SAXS patterns of 9:1 (molar ratio) lipid mixtures used to calculate J_0. The intrinsic curvatures of both DOPE mixtures increased with concentration of the guest lipid (Fig. S1, insert), albeit stronger for lyso-PE, as can be expected from its cone-like molecular shape, due to the difference of projected lateral areas of the PE head and the single hydrocarbon chain (8). The resulting J_0's for all lipids relevant for this study are listed in Tab. S2. In agreement with the qualitative assessment based on the molecular shape we found a significant positive J_0 for lyso-PE, while J_0 of POPG was, within experimental uncertainty, essentially zero. In order to test the effect of buffer on intrinsic lipid curvatures we also measured POPE, which readily forms in the presence of 12 wt.% tricosene a H_{II} phase at 35°C (Fig. S1), see also (6). The derived J_0 = -0.35 nm^{-1} is slightly more negative than our previously reported value. We attribute this to the effect of buffer ions on PE headgroup hydration, which is further supported by a similar decrease of J_0 for DOPE (-0.45 nm^{-1}) as compared to the value found in pure water (6). For the present study such subtleties are, however, of less significance. Here we are interested in estimates for net intrinsic curvatures

of lipid mixtures, which were calculated by their molecular averages upon assuming linear additivity

$$J_0^{mix} = \sum_i \chi_i J_0^{(i)}. \qquad (3)$$

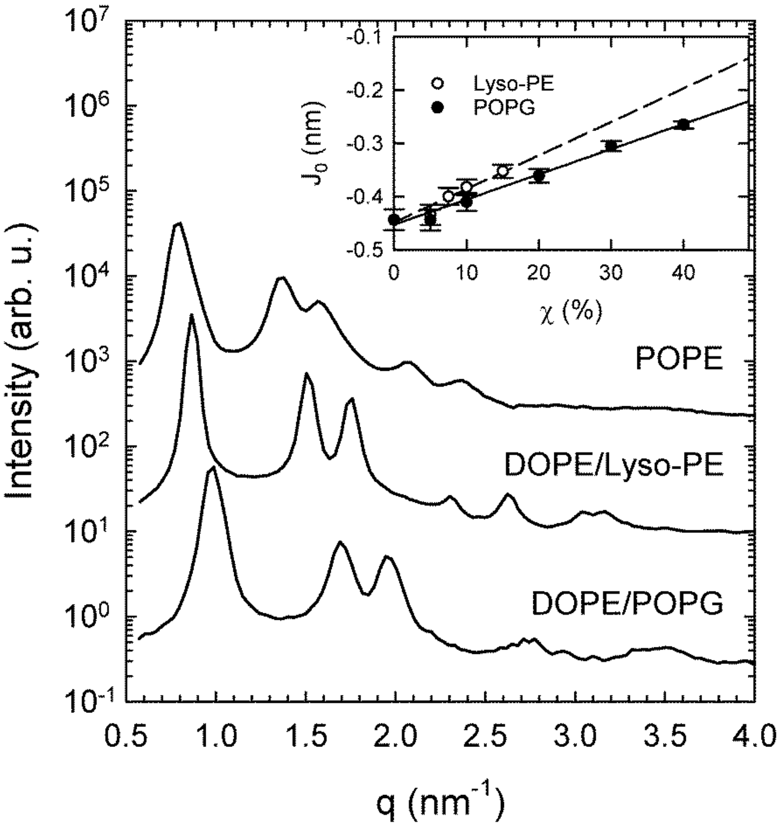

Figure S1: SAXS patterns of DOPE/POPG (9:1 mol/mol), DOPE/lyso-PE (9:1 mol/mol) and POPE at 35°C. All samples contained 12 wt% tricosene. Data are offset vertically for clarity of presentation. The insert shows the change of intrinsic curvatures as a function of POPG and lyso-PE concentration in DOPE, which is used to estimate their J_0's.

Table S2: Intrinsic curvatures of presently studied lipids at 35°C.

**Lipid**	J_0 **[nm^{-1}]**
lyso-PE	0.180 ± 0.123
POPG	0.020 ± 0.030
POPE	-0.350 ± 0.007
POPC*	-0.022 ± 0.010
Chol*	-0.494 ± 0.013

* Data taken from ref. (6)

FLUORESCENCE SPECTROSCOPY - LEAKAGE ASSAY

Leakage of the aqueous content of ANTS/DPX-loaded LUVs upon incubation with peptides was determined according to (9) and as detailed previously (1). Briefly, 2 ml of LUV suspension was filled into quartz cuvettes and equilibrated at 37°C for 5 minutes. Incremental amounts of peptide solutions prepared in Hepes buffer were added to the LUV suspension with µ-pipettes and mixed using a magnetic stirrer to achieve final peptide concentrations ranging from 0.016 - 1 µM, corresponding to peptide-to-lipid molar ratios from 1:3200 to 1:50. Fluorescence emission was recorded 5 minutes before and after the addition of peptides on a Cary Eclipse Fluorescence Spectrophotometer (Varian Inc., now Agilent Technologies, California, US) using an excitation wavelength of λ_{ex} = 360 nm and an emission wavelength λ_{em} = 530 nm. The slit widths for excitation and emission monochromators were set to 10 nm. Percentage of leakage was calculated from the fraction of the leakage (10)

$$I_F = \frac{(F - F_0)}{(F_{max} - F_0)}, \qquad \text{(S4)}$$

where F is the measured fluorescence, F_0 is the initial fluorescence without peptide, and F_{max} is the fluorescence corresponding to 100% leakage gained by addition of 1% Triton X-100.

SODIUM DODECYL SULFATE POLYACRYLAMIDE GEL ELECTROPHORESIS (SDS-PAGE)

In order to verify the dimeric or monomeric state of peptides, 2 µg of peptides were dissolved in loading buffer without DTT and separated by a one-dimensional SDS-PAGE using 5 % stacking and 18 % separating gels according to the method of Laemmli (11). Peptides were stained for 30 min with Coomassie brilliant blue after electrophoretic separation (11). An ultra-low range molecular weight marker was used to estimate the size of peptides after separation via SDS-PAGE. Experiments show that L18W-PGL-GGCa and MG2-GGCa form homodimers in buffer solution (pH ~ 7) (Fig. S2), in agreement with (12). Application of DTT leads to a drop of peptide activity to levels observed for single peptides, consistent with monomer formation (Fig. S3).

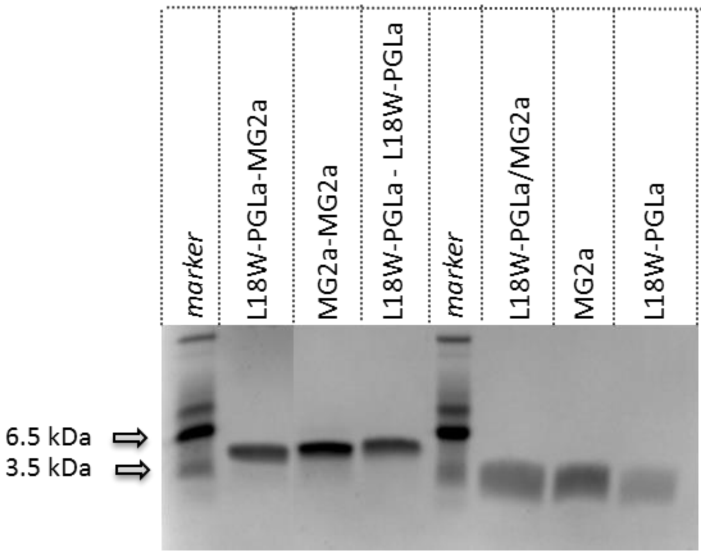

Figure S2: SDS-PAGE verifying dimeric and monomeric forms of magainins

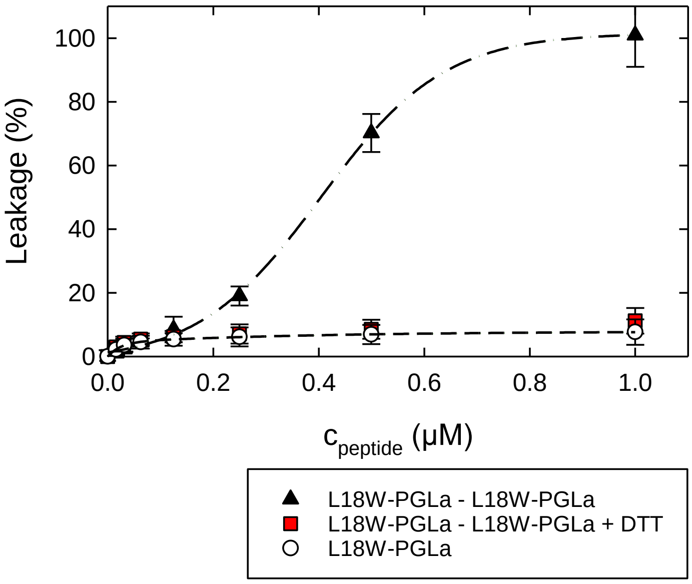

Figure S3: Effect of DTT addition to L18W-PGLa homodimers on leakage of POPE/POPG 3:1 mol/mol LUVs. For comparison, leakage induced by L18W-PGLa is shown as well (see also Fig. 2 *B*). The lipid concentration was 50 µM. Lines are guides to the eye.

MC SIMULATIONS

Monte Carlo simulations were performed using the Metropolis scheme and computationally efficient implicit solvent coarse-grained models. Sizes and distances of the models were calculated in reduced units, which (based on real particle dimensions) roughly translate to nm. The studied systems were composed of 200 lipid molecules and a single peptide. Lipids were

described by a three-bead model developed by Cooke and Deserno (13), using one bead for the head group of diameter d_h and two beads for tails each of diameter d_t (Fig. S4 *A*).

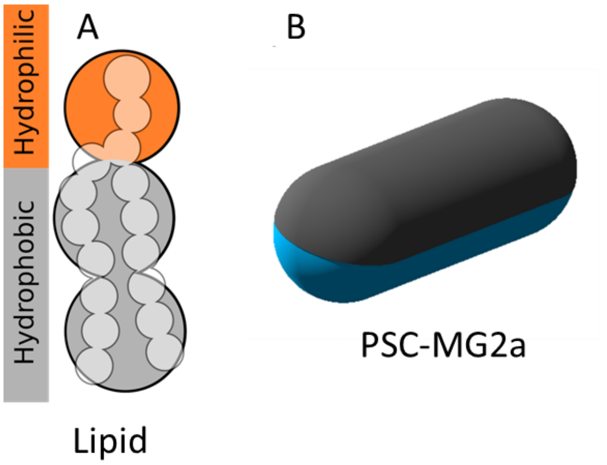

Figure S4: Schematic representations of the employed models. Panel A: Three-bead lipid model with lipid mapping (shown as silhouette). Panel B: PSC model of MG2a. Hydrophobic patches/regions are represented in grey, while orange and blue depict hydrophilic parts of lipid and peptide, respectively.

Three lipids with different intrinsic curvatures were simulated by variation of headgroup size (Fig. S5). The size of head bead affects J_0. Assuming cylindrical geometry of lipids one can calculate the intrinsic curvature by (14)

$$J_0 = \frac{2}{l}\left(1 - \frac{V}{A_h l}\right), \tag{S5}$$

where l is the length of the hydrophobic tails, A_h is the effective headgroup area and V is the hydrocarbon chain volume. However, there are more possibilities to calculate the l and $V/(A_h l)$. The first possibility is based on the geometry of individual molecules with $A_h = \pi d_h^2/4$, $V = \pi d_t^2 l/4$, and $l = 2d_t$. For $d_h = 0.85$ nm, 0.95 nm and 1.05 nm, this results for $d_t = 1$ nm in $J_0 = -0.38$ nm^{-1}, -0.11 nm^{-1}, and $+0.09$ nm^{-1}, respectively. However, this considers only excluded volume interactions between lipid molecules in ideal configuration. We therefore adhere to the second possibility, which derives J_0 from MC simulations and consequently include also contributions from temperature and the lipid parametrization (bonds, bending, and tail attraction). In this case the length of hydrophobic tails is calculated from the average distance of headgroup beads from the bilayer center, i.e. $d_l = 2.5$ nm. The headgroup areas are derived from the simulation box size of the equilibrated membrane at zero tension, which yields $A_h = 1.025$ nm^2, 1.145 nm^2 and 1.215 nm^2 with increasing d_h. Assuming that $A_h \propto d_h^2$ we can interpolate the headgroup area to perfectly cylindrical lipids (i.e., $d_h = d_t$ and $J_0 = 0$), yielding $A_h^0 = 1.176$ nm^2. Consequently Eq. (S5) is modified to

$$J_0 = \frac{2}{l}\left(1 - \frac{A_h^0}{A_h}\right), \quad \text{(S6)}$$

leading to $J_0 = -0.12$ nm^{-1}, -0.02 nm^{-1}, and $+0.03$ nm^{-1} for $d_h = 0.85$ nm, 0.95 nm and 1.05 nm, respectively. The lipid with the largest headgroup leads to $J_0 > 0$.

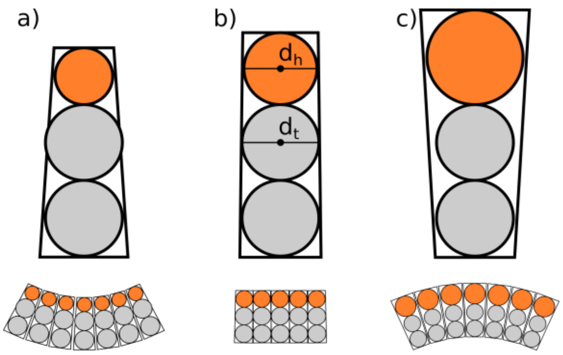

Figure S5: Schematic representation of lipid shape and induced curvature. Diameters of lipid heads and tails are denoted as d_h and d_t, respectively, with d_t = 1 nm. Conical lipids a) and c) had d_h = 0.85 and 1.05 nm, respectively. For cylindrical lipids b) d_h = 0.95 nm. The corresponding intrinsic curvatures are a) J_0 = -0.12 nm^{-1}, b) J_0 = -0.02 nm^{-1}, and c) J_0 = +0.03 nm^{-1}.The induced curvature is shown below each lipid. Differences in headgroup sizes are magnified for clarity.

The peptide was modelled by a patchy spherocylinder (PSC) (15), setting the peptide length to 4 nm and its diameter to 1 nm to roughly match the size and polarity of MG2a, i.e. half of the peptide surface was hydrophobic (Fig. S4 *B*). At either side of the patch, there is a 5° switching range to linearly scale the interaction potential to zero. The remaining surface is purely repulsive and thus considered to be hydrophilic. For more details on the model, see (15).

To mimic the hydrophobic effect, effective tail-tail interactions were used to drive membrane self-assembly leading to an effective attractive potential with a cos^2 profile. Further, the same potential was used to describe interactions between lipid tails and the peptide's hydrophobic patch. Repulsive interactions were modeled by Weeks-Chandler-Anderson (shifted and truncated Lennard-Jones) potential. The PSC-MG2a switching range between hydrophobic and hydrophilic surfaces was set to 1.0 nm. As described in the original article, the switching range of lipids was extended to 1.6 nm to keep the membrane in the fluid phase (13). The depth of the attractive potential well was -1 kT (k is the Boltzmann constant and T is the temperature) for lipid-tail particles and -1 kT per unit length of PSC line segment.

The membrane bilayer was assembled in the XY-plane of a rectangular box. The initial box size was about $11 \times 11 \times 50$ nm. Periodic boundary conditions were applied in all three

dimensions, forming “infinite” membrane plane. Simulations were performed under constant temperature and number of particles, while keeping zero lateral tension in the XY-plane. We used standard MC trial moves to move the peptide/lipid beads/whole lipids and to modify the box size (16). All simulations were carried out using our in-house software (freely available github.com/robertvacha/SC).

Free energy calculations for peptide translocation were performed using the Wang-Landau method (17). The free energy surface was considered converged once the modification factor was below 10^{-7}. Subsequently, simulations maintaining detailed balance of peptide translocation were performed and the obtained free energy surface was used as an external bias. The obtained histogram was then used to further improve the previously calculated free energy surface. The error of the free energy profiles is about 1 kT.

For proper sampling of peptide insertion, two collective variables (CVs) were used. First CV is the distance between peptide and the membrane center of mass. The free energy profile is symmetrical around the membrane center and asymmetry shows the calculation error. Second CV is the peptide orientation with respect to the membrane normal. The orientation is expressed as the cosine of the angle. Change of orientation from perpendicular to parallel (with respect to the membrane normal) is considered. For clarity, the second collective variable is averaged out and only the dependence on peptide position is shown.

IN VITRO ASSAY FOR AMP ACTIVITY

To study the effect of magainins on metabolically active bacteria, we assessed the antimicrobial activity of the peptides by determining the MIC values according to Clinical and Laboratory Standards Institute (CLSI, formerly National Committee for Clinical Laboratory Standards) guidelines.

Inhibition of bacterial growth by frog peptides was determined using an automated turbidimetric-based system (Bioscreen C, Labsystem, Helsinki, Finland), which measures absorbance of cell cultures in 100-well plates at regular time intervals (λ = 580 nm). Assays were performed in Mueller-Hinton (MH) broth without Ca^{2+} (18) using Bioscreen polystyrene honeycomb 100-well plates. Briefly, serial dilutions of the AMPs or control antibiotics (gentamicin and ampicillin B) were made in MH broth and dispensed with a multi-pipette into

polystyrene honeycomb 100-well plates. *E. coli* K12 cells grown for 18–20 h on agar plates were suspended in saline adjusting the turbidity to an optical density at λ=565 nm (OD_{565}) of 0.5 (~1×10^8 cells/mL) using a DEN-1B densitometer (Grant Instruments, Cambridge, UK). A fifty-fold dilution of this *E. coli* suspension prepared in MH broth was mixed with 50 µL (1:1 volume ratio) of a given peptide solution by three times up and down pipetting. Resulting cell suspensions were grown at 37°C with continuous, low amplitude shaking. The turbidity was measured in intervals of 5 minutes (filter bandwidth: λ= 420 – 580 nm). MIC was considered as the minimum concentration of peptide that inhibited any increment of turbidity after 48 h.

REFERENCES

1. Zweytick, D., G. Deutsch, J. Andrä, S. E. Blondelle, E. Vollmer, R. Jerala, and K. Lohner. 2011. Studies on lactoferricin-derived Escherichia coli membrane-active peptides reveal differences in the mechanism of N-acylated versus nonacylated peptides. J Biol Chem 286:21266–21276.
2. Broekhuyse, R. M. 1968. Phospholipids in tissues of the eye. I. Isolation, characterization and quantitative analysis by two-dimensional thin-layer chromatography of diacyl and vinyl-ether phospholipids. Biochim Biophys Acta 152:307–315.
3. Buboltz, J. T., and G. W. Feigenson. 1999. A novel strategy for the preparation of liposomes: rapid solvent exchange. Biochim Biophys Acta 1417:232–245.
4. Rieder, A. A., D. Koller, K. Lohner, and G. Pabst. 2015. Optimizing rapid solvent exchange preparation of multilamellar vesicles. Chemistry and physics of lipids 186:39–44.
5. Alley, S. H., O. Ces, M. Barahona, and R. H. Templer. 2008. X-ray diffraction measurement of the monolayer spontaneous curvature of dioleoylphosphatidylglycerol. Chemistry and physics of lipids 154:64–67.
6. Kollmitzer, B., P. Heftberger, M. Rappolt, and G. Pabst. 2013. Monolayer spontaneous curvature of raft-forming membrane lipids. Soft Matter 9:10877.
7. Leikin, S., M. M. Kozlov, N. L. Fuller, and R. P. Rand. 1996. Measured effects of diacylglycerol on structural and elastic properties of phospholipid membranes. Biophys J 71:2623–2632.
8. Israelachvili, J. N. 2011. Intermolecular and surface forces, 3rd ed. Academic Press, Burlington, MA.
9. Ellens, H., J. Bentz, and F. C. Szoka. 1985. H+- and Ca2+-induced fusion and destabilization of liposomes. Biochemistry 24:3099–3106.
10. Ladokhin, A. S., W. C. Wimley, and S. H. White. 1995. Leakage of membrane vesicle contents: Determination of mechanism using fluorescence requenching. Biophys J 69:1964–1971.

11. Laemmli, U. K. 1970. Cleavage of structural proteins during the assembly of the head of bacteriophage T4. Nature 227:680–685.

12. Hara, T., Y. Mitani, K. Tanaka, N. Uematsu, A. Takakura, T. Tachi, H. Kodama, M. Kondo, H. Mori, A. Otaka, F. Nobutaka, and K. Matsuzaki. 2001. Heterodimer formation between the antimicrobial peptides magainin 2 and PGLa in lipid bilayers: a cross-linking study. Biochemistry 40:12395–12399.

13. Cooke, I. R., K. Kremer, and M. Deserno. 2005. Tunable generic model for fluid bilayer membranes. Phys Rev E 72:11506.

14. Marsh, D. 1996. Intrinsic curvature in normal and inverted lipid structures and in membranes. Biophys J 70:2248–2255.

15. Vácha, R., and D. Frenkel. 2011. Relation between molecular shape and the morphology of self-assembling aggregates: a simulation study. Biophys J 101:1432–1439.

16. Frenkel, D., and B. Smit. 2002. Understanding Molecular Simulation. Academic Press, San Diego, CA.

17. Wang, F., and D. P. Landau. 2001. Efficient, multiple-range random walk algorithm to calculate the density of states. Phys Rev Lett 86:2050–2053.

18. Mueller, J. H., and J. Hinton. 1941. A protein-free medium for primary isolation of the gonococcus and meningococcus. Proc Soc Exp Biol Med 48:330–333.
